# Identification of integrative and conjugative elements in pathogenic and commensal *Neisseriaceae* species via genomic distributions of DNA uptake sequence dialects

**DOI:** 10.1099/mgen.0.000372

**Published:** 2020-05-04

**Authors:** Alex Hughes-Games, Adam P. Roberts, Sean A. Davis, Darryl J. Hill

**Affiliations:** ^1^​ School of Cellular and Molecular Medicine, University of Bristol, Bristol, UK; ^2^​ Bristol Centre for Functional Nanomaterials, HH Wills Physics Laboratory, University of Bristol, Bristol, UK; ^3^​ Centre for Drugs and Diagnostics, Department of Tropical Disease Biology, Liverpool School of Tropical Medicine, Liverpool, UK; ^4^​ School of Chemistry, University of Bristol, Bristol, UK

**Keywords:** integrative and conjugative elements, *Neisseria*, DNA uptake sequence, commensal neisseria, mobile genetic elements, horizontal gene transfer

## Abstract

Mobile genetic elements (MGEs) are key factors responsible for dissemination of virulence determinants and antimicrobial-resistance genes amongst pathogenic bacteria. Conjugative MGEs are notable for their high gene loads donated per transfer event, broad host ranges and phylogenetic ubiquity amongst prokaryotes, with the subclass of chromosomally inserted integrative and conjugative elements (ICEs) being particularly abundant. The focus on a small number of model systems has biased the study of ICEs towards those conferring readily selectable phenotypes to host cells, whereas the identification and characterization of integrated cryptic elements remains challenging. Even though antimicrobial resistance and horizontally acquired virulence genes are major factors aggravating neisserial infection, conjugative MGEs of *Neisseria gonorrhoeae* and *Neisseria meningitidis* remain poorly characterized. Using a phenotype-independent approach based on atypical distributions of DNA uptake sequences (DUSs) in MGEs relative to the chromosomal background, we have identified two groups of chromosomally integrated conjugative elements in *Neisseria *: one found almost exclusively in pathogenic species possibly deriving from the genus *Kingella *, the other belonging to a group of *Neisseria mucosa *-like commensals. The former element appears to enable transfer of traditionally gonococcal-specific loci such as the virulence-associated toxin–antitoxin system *fitAB* to *N. meningitidis* chromosomes, whilst the circular form of the latter possesses a unique attachment site (*attP*) sequence seemingly adapted to exploit DUS motifs as chromosomal integration sites. In addition to validating the use of DUS distributions in *Neisseriaceae* MGE identification, the >170 identified ICE sequences provide a valuable resource for future studies of ICE evolution and host adaptation.

## Data Summary

A subset of genome sequences analysed in this work were obtained from the GenBank database and are available at https://www.ncbi.nlm.nih.gov/assembly/ by searching for the following accession numbers: *Neisseria subflava* (GCA_001836705.1 and GCA_001815375.1); *Kingella negevensis* SW7208426 (GCA_900177895.1); *Neisseria gonorrhoeae* FA19 (GCA_001047225.1), MS11 (GCA_000156855.2), WHO F (GCA_900087635.2); *Neisseria meningitidis* MC58 (GCA_000008805.1), NM442 (GCA_000328265.2); *Neisseria mucosa* ATCC 25996 (GCA_000173875.1), HMSC055H02 (GCA_001813335.1); *Neisseria sicca* C2007003584 (GCA_003044425.1); *Neisseria* sp. 1273_NMEN (GCA_001062585.1); *Simonsiella muelleri* ATCC 29453 (GCA_000163775.2); *Alysiella crassa* DSM 2578 (GCA_000745955.1); *Neisseria elongata* M15910 (GCA_003351685.1); *Neisseria iguanae* (GCA_003013245.1); *Neisseria zoodegmatis* (GCA_900187305.1). All other *N. meningitidis *, *N. gonorrhoeae* and *Neisseria lactamica* sequences analysed herein are available from the PubMLST database at https://pubmlst.org/neisseria by searching the isolate database with the ID numbers given in Table S1 (available with the online version of this article). Two integrative and conjugative elements (ICEs) with experimentally confirmed mobility, ICE*riNK* of *N. meningitidis* isolate 61 951 and ICE*Nmu* of *N. mucosa* ATCC 25996, were allocated the names Tn*6744* and Tn*6745*, respectively, by The Transposon Registry, and corresponding registry entries can be viewed at https://transposon.lstmed.ac.uk/. The Python script for detecting putative genomic islands and supporting files are available from https://github.com/alexh-g.

**Impact Statement**

The self-transmissible mobile genetic elements termed integrative and conjugative elements (ICEs) are major drivers of microbial gene transfer that can exacerbate disease severity and cause antibiotic failure. Detection of ICEs by *in vitro* methods can be challenging, with *in silico* genome analysis providing a more reliable approach. Here, we establish a method based on the genomic distributions of specific sequence motifs to detect ICEs within the family *Neisseriaceae *, which contains the globally important pathogens *Neisseria gonorrhoeae* and *Neisseria meningitidis *. Using this technique, diverse sequence variants of an ICE family were shown to be widespread amongst these two pathogens and which could have originated in the commensal *Kingella negevensis *, paving the way for future studies of epidemiological associations and ICE host adaptation. A separate ICE family amongst commensal *Neisseria* was also identified and gives insights into ICE evolution via an apparent strategy for repurposing and exploiting genomic hallmarks of the neisserial self-specific DNA-uptake system.

## Introduction

The human microbiota contains ~8–17 harmless commensal species of the genus *Neisseria* [1], but the two close relatives *Neisseria meningitidis* and *Neisseria gonorrhoeae* underlie major public-health challenges. *N. meningitidis* generally exists as a commensal colonizing the upper respiratory tract; however, invasive strains can cause life altering, or even fatal, meningococcal disease. *N. gonorrhoeae* is an obligate human pathogen causing the sexually transmitted infection (STI) gonorrhoea, one of the most prevalent STIs with 80–100 million new cases worldwide each year [2–4]. Both pathogenic species are constitutively competent for transformation, enabling the acquisition of novel genetic material from naked DNA in the extracellular milieu and exacerbating pathogenicity via horizontal acquisition of antibiotic resistance and virulence factors [5]. Unlike many other naturally transformable prokaryotes, meningococci and gonococci exhibit a strong preference for uptake and transformation of conspecific genomic DNA or that of close relatives due to recognition of highly overrepresented ~12 bp nucleotide motifs termed DNA uptake sequences (DUSs) [6, 7]. Similar self-specific DNA uptake systems have been demonstrated for several species of the family *Neisseriaceae* across three genera [8, 9], but separate phylogenetic groups were shown to employ distinct uptake sequence variants known as dialects [10]. Reduced levels of uptake and transformation are observed for exogenous DNA containing heterologous DUS dialects compared to DNA possessing the native dialect of recipient cells [10, 11]. Eight unique dialect sequences have been identified to date, all of which contain a conserved 5ʹ-CTG-3ʹ core, but differ by up to 6 bases in flanking positions [10]. Many examples of clinically important cross-species gene transfer within compatible dialect groups have been highlighted, such as exchange of the *mtr* [12] efflux pump and *penA* [13] penicillin-binding protein resistance alleles between commensal *Neisseria* spp. and *N. gonorrhoeae *, or acquisition of a gonococcal denitrification gene cassette by meningococcal urethritis isolates [14].

The majority of research into horizontal gene transfer in *Neisseria* has focussed on this unique self-specific system for natural transformation, but despite the barriers to interspecific gene transfer imposed by the neisserial DNA uptake system, DUS-independent gene transfer can occur via transduction and conjugation, where DUS-selective DNA translocation by type IV pili is supplanted by transfer via bacteriophage and type IV secretion systems (T4SSs). For example, several gonococcal and meningococcal prophages have been identified [15, 16], as well as three conjugative plasmids of *N. gonorrhoeae* [17]. The functional roles of these prophages have been relatively well studied [15, 18–23]; however, research on neisserial conjugative mobile genetic elements (MGEs) and their phenotypic consequences is lacking [17]. In particular, very little attention has been given to chromosomal conjugative MGEs such as integrative and conjugative elements (ICEs) in pathogenic *Neisseria *, despite their clinical importance as a conduit for antimicrobial resistance and virulence factors in other genera and ubiquity amongst prokaryotes in general [24]. ICEs are modular elements between 18 and 500 kb in size bearing a core gene set encoding a T4SS-based conjugation apparatus and an integrase or resolvase mediating their genomic integration and excision, as well as a set of accessory genes that may confer selective advantages to the host. Recombination and transposition events are prevalent in ICEs, enabling acquisition of chromosomal, plasmid and transposon-borne loci, and in some cases leading to the formation of large multidrug-resistance modules [25–27]. Under normal conditions, ICEs are integrated into the host genome and remain in a transcriptionally repressed dormant state, but may be excised into a circular form and de-repressed in response to factors such as DNA damage, secreted signalling molecules or exposure to antimicrobials [28]. The majority of ICEs studied to date provide clear benefits to the host, such as antibiotic resistance or exploitation of alternative carbon sources, but it is not clear whether this is a general feature of ICEs or results from difficulties in identifying and functionally analysing MGEs in the absence of conveniently selectable phenotypes [28]. Thus, bioinformatic methods that identify ICEs independently from such phenotypes are desirable to ensure cryptic elements are not under-represented.

Chromosomally integrated MGEs transferred via conjugation and transduction are typically located on relatively large genomic islands (GIs; defined here as putative horizontally acquired chromosomal regions with atypical nucleotide *k*-mer composition) >10 kb as a result of their self-encoded transfer machinery. GIs are often identified in genome sequencing data via a combination of nucleotide composition and/or codon usage that differs from the bulk genome, proximity to phage- or conjugation-associated genes, and homology with putative donor genomes, but the distribution of DUSs in *Neisseriaceae* genomes provides an additional indicator of exogenous DNA. It is important to note that although most neisserial genomes contain a dominant DUS dialect (typically with 2000–6000 copies), they often contain significant numbers of alternative dialect motifs. To avoid confusion, we will refer to the single most abundant dialect in a genome as the primary DUS (prDUS) and all other neisserial dialects found in the same chromosome as ancillary DUSs (anDUSs). The mean proportion of genome occupied by prDUSs nucleotides typically ranges from 1 to 2.5 % [10], corresponding to roughly one site every 400–1100 bp, but drastic reductions in DUS density are often found in meningococcal [29] and gonococcal [30] GIs. Crucially for the work presented here, in the case of GIs originating within *Neisseriaceae *, the anDUS dialect content of the GI itself can be used to infer the corresponding dialect group of the original donor species.

Due to the highly modular nature of ICEs and the presence of repetitive elements, such as transposons, short-read sequencing methods often occlude their accurate assembly or result in misidentification as plasmids; however, the increasing assembly quality and abundance of available *Neisseriaceae* genome sequences prompted us to search for previously unidentified large chromosomally located MGEs. Using the chromosomal distribution of DUSs as an indicator of horizontal acquisition from other *Neisseriaceae* donors, we identified several uncharacterized groups of GIs in neisserial pathogens and commensals. Two groups of islands were selected for further characterization due to their large size, clear delimitation and abundance in sequencing data, and were both identified as ICEs carrying a variety of toxin–antitoxin (TA) systems, transposons and numerous predicted ORFs for uncharacterized proteins. Active excision of both ICEs was indicated by PCR amplification using primers specific to the predicted circular form *attP* region. In one case, sequencing of these amplicons revealed a DUS of the host genome present at the attachment site, suggesting an interesting evolutionary strategy employed by neisserial ICEs to expand their host range among DUS dialect groups via *attP* mutations.

## Methods

### GI detection and analysis

Putative GIs were identified using a custom Python script which, for a given GenBank format genome sequence file input, carries out the following steps: (i) detects and annotates all occurrences of the eight 12 bp DUS dialects previously outlined by Frye *et al*. [10] (using the emboss Fuzznuc module [31]); (ii) identifies the prDUS and annotates large gaps between adjacent prDUS sites if they are above a specified bp threshold calculated based on the typical prDUS distribution across the entire genome; (iii) annotates accessory genomic elements (AGEs) detected using AGEnt by referencing a core genome file calculated in advance with Spine [32] (this step was omitted in cases where a lack of available sequences prohibited construction of a core genome); (iv) produces a DNAPlotter [33] template file for visualizing the resulting annotations. The purpose of identifying AGEs was to validate the use of local prDUS under-representation as a proxy for GIs, and to help distinguish between islands belonging to core and accessory genomes. Core genomes for *N. meningitidis* and *N. gonorrhoeae* were both calculated from 15 complete genomes. For the work presented here, the threshold for ‘large’ prDUS gap annotation was picked as four times the interquartile range for all genomic prDUS gaps, which gave a good balance between false positives and negatives when compared with AGEs detected with AGEnt. Synteny comparisons were performed with local blastn [34] and visualized with the Artemis Comparison Tool [35]. All genome sequences were sourced from GenBank or PubMLST [36], and species assignments for erroneous or unspecified cases were determined using the PubMLST ribosomal multilocus sequence typing (rMLST) server [37]. It has been suggested that *Neisseria sicca* should be considered the same species as *Neisseria mucosa* [38], but the GenBank entry for *N. sicca* C2007003584 (accession no. GCA_003044425.1) was only predicted to be *N. mucosa* with 38 % confidence by rMLST; hence, the original designation was retained in this work. PubMLST rMLST was also unable to definitively assign any species to *Neisseria* sp. 1273_NMEN [GenBank accession no. GCA_001062585.1; erroneously listed as *N. meningitidis* in National Center for Biotechnology Information (NCBI) databases], but identified *N. mucosa* as the closest known relative. Incomplete genomes were concatenated prior to analysis and sequences with poorly annotated ICE regions were re-annotated via the RASTtk pipeline [39]. Relaxase MOB family assignments were made using the MOBscan server (https://castillo.dicom.unican.es/mobscan/) [40].

Clustering of gonococcal and meningococcal ICE variants was determined from a Neighbour-Net [41] network plotted in SplitsTree4 [42]. The distance matrix input file for SplitsTree4 was produced by comparison of allele values from the PubMLST ‘VirB T4SS’ scheme using the BIGSdb Genome Comparator tool [43]. Branch lengths, therefore, reflect the number of VirB T4SS loci with different allele sequences between isolates, as well as locus presence or absence.

### PCR and sequencing of excised circular ICEs

*N. mucosa* ATCC 25996 and *N. meningitidis* carrier isolates (PubMLST IDs 61951, 61970, 61957 and 61975) were grown for 16–18 h on gonococcal agar base plates at 37 °C in 5 % CO_2_. Genomic and plasmid DNA were extracted using a DNeasy blood and tissue kit and a QIAprep spin miniprep kit (Qiagen), respectively, according to the manufacturer’s instructions. Extracted DNA was used as a template in PCR amplifications with differing combinations of primers NmeICE-F1 (5ʹ-GAAATCTTAAGCCAAGGCAG-3ʹ), NmeICE-F2 (5ʹ-CATTCAAGACGACATCAGC-3ʹ) and NmeICE-R (5ʹ-GTTGTGCCAAATCGTTAAATC-3ʹ) for *N. meningitidis* or MucICE-F1 (5ʹ-CTTTGCATATCGGCATATTG-3ʹ), MucICE-F2 (5ʹ-GACAAGCCCAAAGTCCTGTG-3ʹ) and MucICE-R (5ʹ-GGAAAATATCCCATTCTGCATAC-3ʹ) for *N. mucosa *. All reactions used CloneAmp HiFi PCR premix (Takara), 35 cycles with a 57.4 °C annealing temperature and 15 s extension step, and were analysed by gel electrophoresis in a 1 % agarose Tris/borate/EDTA gel. DMSO was added to 8 % (v/v) for reactions with primers NmeICE-F2 and NmeICE-R. Amplified products corresponding to the *attP* region of circular ICEs were purified using a QIAquick PCR purification kit (Qiagen) according to the manufacturer’s instructions, and sequenced by the Source BioScience Sanger sequencing service with the same primers used for amplification.

## Results

### DUS dialect mapping and GI detection

**Fig. 1.**
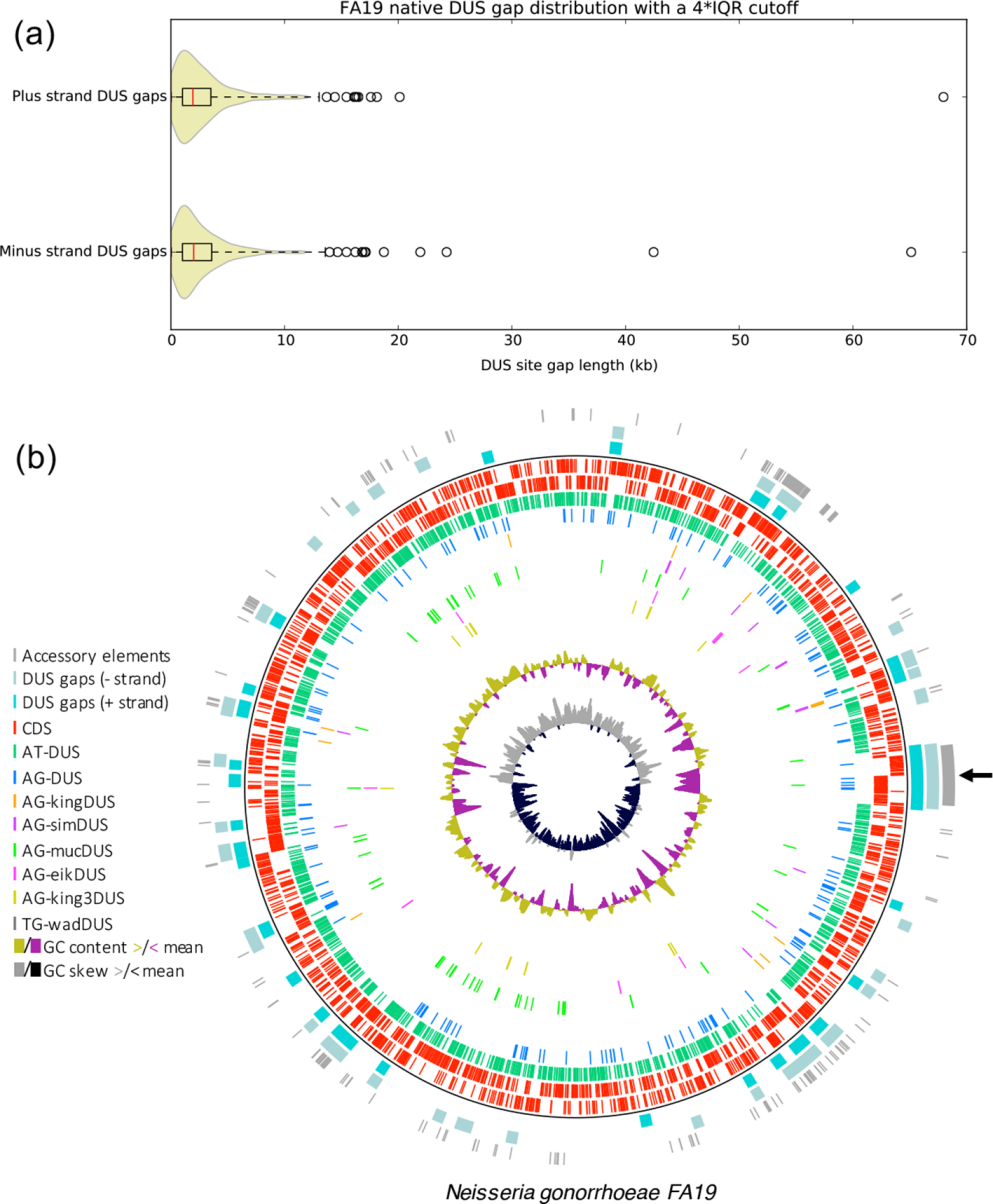
Typical output of a custom Python script combining Fuzznuc and AGEnt to identify horizontally acquired GIs in *Neisseriaceae* genomes and their DUS dialect group of origin. (a) Violin plot showing the distribution of spacing between adjacent prDUS sites for both strands of the gonococcal FA19 strain genome. Circular markers indicate gap lengths exceeding the outlier threshold that are considered GI candidates. The inset black rectangle represents the interquartile range (IQR) with the median idicated by the red line. (b) DNAplotter genomic plot of the FA19 chromosome after processing and annotation via the Python script. Positions of coding regions and DUS sites are indicated on tracks within the black ring, whereas outer tracks correspond to AGEs and large tracts where the native DUS is absent. The well-studied GGI located at the replication terminus is indicated by an arrow. Incomplete genomes were concatenated prior to running the script, so G+C skew is plotted as a rough indicator of genome assembly level. Coding sequences (CDS) are shown as red bars. DUS dialect nomenclature and sequences are outlined in Table S2.

The lack of prDUSs in GIs has been noted previously [29, 30], but we sought to identify chromosomal regions in which prDUSs are under-represented and anDUSs are overrepresented, so as to expedite identification of potential donor species. This approach is based on the principle that high anDUS density within horizontally acquired regions is likely to reflect their prolonged coevolution with a previous host species bearing the same DUS dialect. In order to identify GI candidates, complete or concatenated whole-genome sequences were passed through a custom Python script that detects each exact match of the eight DUS dialect motifs defined in the reference by Frye *et al*. [10]. The distribution of gap lengths between adjacent prDUSs is then determined separately for both sequence strands and used to establish outliers above a threshold gap size that are considered candidate GI-containing regions. Gap distributions for gonococcal strain FA19 are shown in Fig. 1a and are typical of all pathogenic *Neisseria* analysed, which exhibit a positive skew and median size of 1.9–2.1 kb. All dialect motifs and outlier prDUS gaps were also annotated and plotted by the script to produce genome maps highlighting these features (Fig. 1b). Plots were manually inspected for putative GIs with an overrepresentation of anDUSs, which were then assessed for their possible function and species of origin.

Several previously characterized GIs were highlighted by this method, the largest and most studied of which is known as the gonococcal genetic island (GGI), which is found in ~80 % of *N. gonorrhoeae* isolates and has been associated with antimicrobial resistance and disseminated gonococcal infection [44, 45]. Consistent with its horizontal acquisition, the GGI typically contains no copies of the prDUS (AT-DUS: 5ʹ-ATGCCGTCTGAA-3ʹ; dialect sequence nomenclature is summarized in Table S2), but does possess several AG-DUS sites (5ʹ-AGGCCGTCTGAA-3ʹ), suggesting that it was hosted by species within the AG-DUS dialect group prior to acquisition by the gonococcus. blast searches of *N. gonorrhoeae* GGI genes against all available AG-DUS-group *Neisseriaceae* produced maximum hits corresponding to two genome sequences (GenBank accession numbers GCA_001836705.1 and GCA_001815375.1) assigned to *Neisseria subflava* by rMLST. Synteny comparisons of gonococcal strain MS11 with both *N. subflava* genomes revealed conserved regions spanning 43 of the 61 GGI ORFs with a mean nucleotide identity of 90 % Fig. S1, indicating this species as a likely donor of the clinically relevant GI. The transfer of disease-associated virulence factors from this commensal to pathogenic *Neisseria* is consistent with a recent study indicating horizontal acquisition of meningococcal capsule locus components from *N. subflava* [46].

**Fig. 2.**
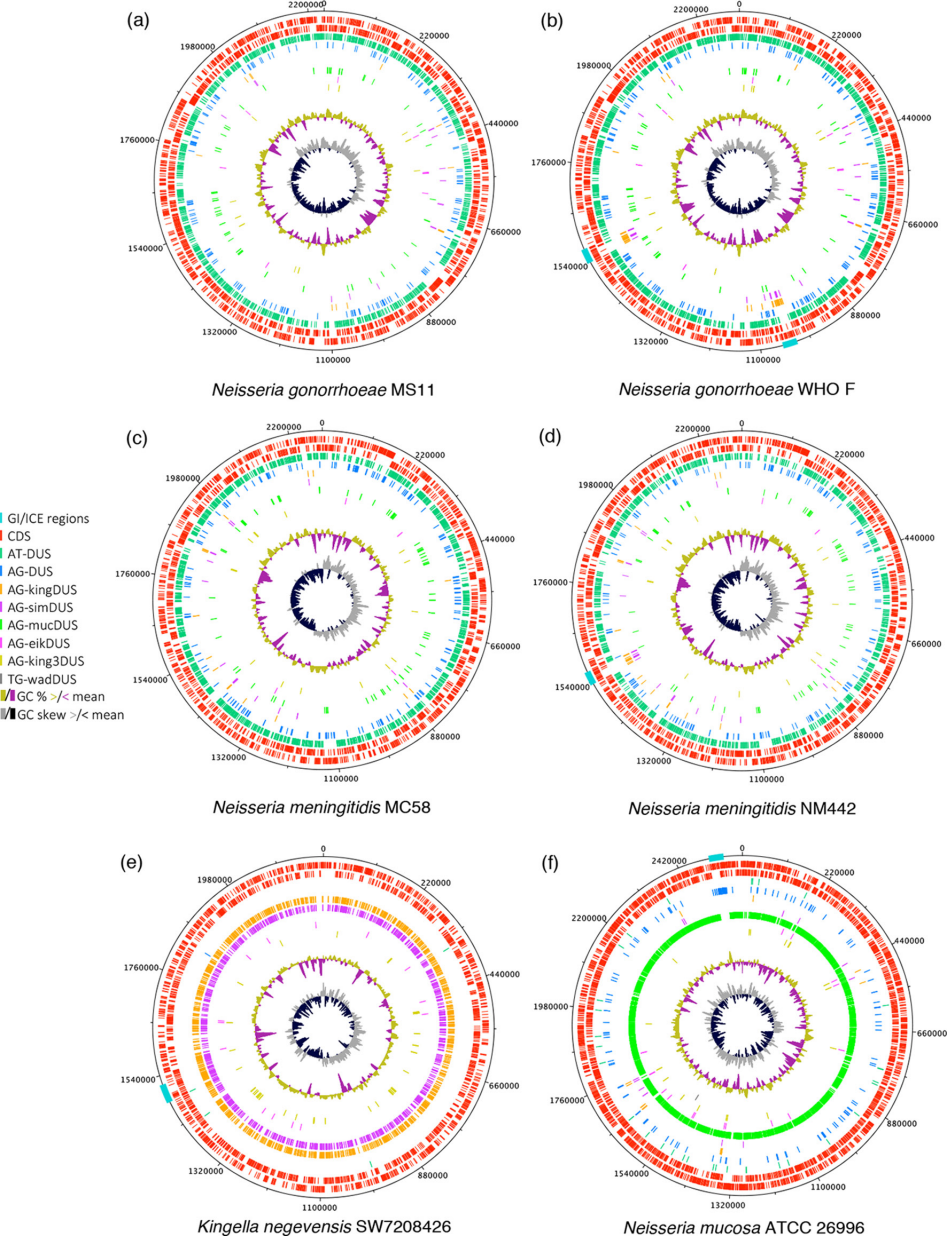
Genomic maps highlighting *Neisseriaceae* GIs deviating in DUS dialect relative to the bulk genome and harbouring ICEs. (a, b) Comparison of gonococcal strain MS11, which contains no AG-king/simDUS dyad islands >10 kb, with strain WHO F, which possesses two duplicated 30 kb ICE regions with numerous AG-king/simDUS sites. (c, d) A similar comparison of meningococcal genomes highlighting the insertion of an AG-king/simDUS island in *N. meningitidis* strain NM442 relative to MC58. (e) *K. negevensis* genome with a DUS profile closely matching that of the ICE regions in b and d. The highlighted segment has matching synteny and high homology to the ICE regions in b and d. (f) Another ICE region with atypical DUS content of AG-DUS relative to the native AG-mucDUS in the genome of *N. mucosa *. Coding sequences (CDS) are shown as red bars.

Another abundant group of GIs highlighted by this technique consists of two-partner secretion islands, which encode polymorphic toxin systems such as the recently characterized *mafB-mafI* modules [47]. However, due to their extensive genetic variation and relatively small size, these islands are difficult to precisely delineate and are not always readily detected via their aberrant DUS distribution. In contrast, several genomic sequences bore much larger (up to 36 kb) GIs that almost exclusively contained anDUSs of a single dialect type and possessed boundaries that could be clearly defined by comparison with genomes lacking the island. This is exemplified by contrasting genomic plots in Fig. 2a, c (lacking GIs) with Fig. 2b, d, respectively. One group of islands was of particular interest due to their abundance in pathogenic *Neisseria* and is analysed in the following section, whereas the final section describes a distinct but structurally similar GI found in the commensal *N. mucosa* and its close relatives.

### Characterization of a pathogenic *Neisseria* ICE

**Fig. 3.**
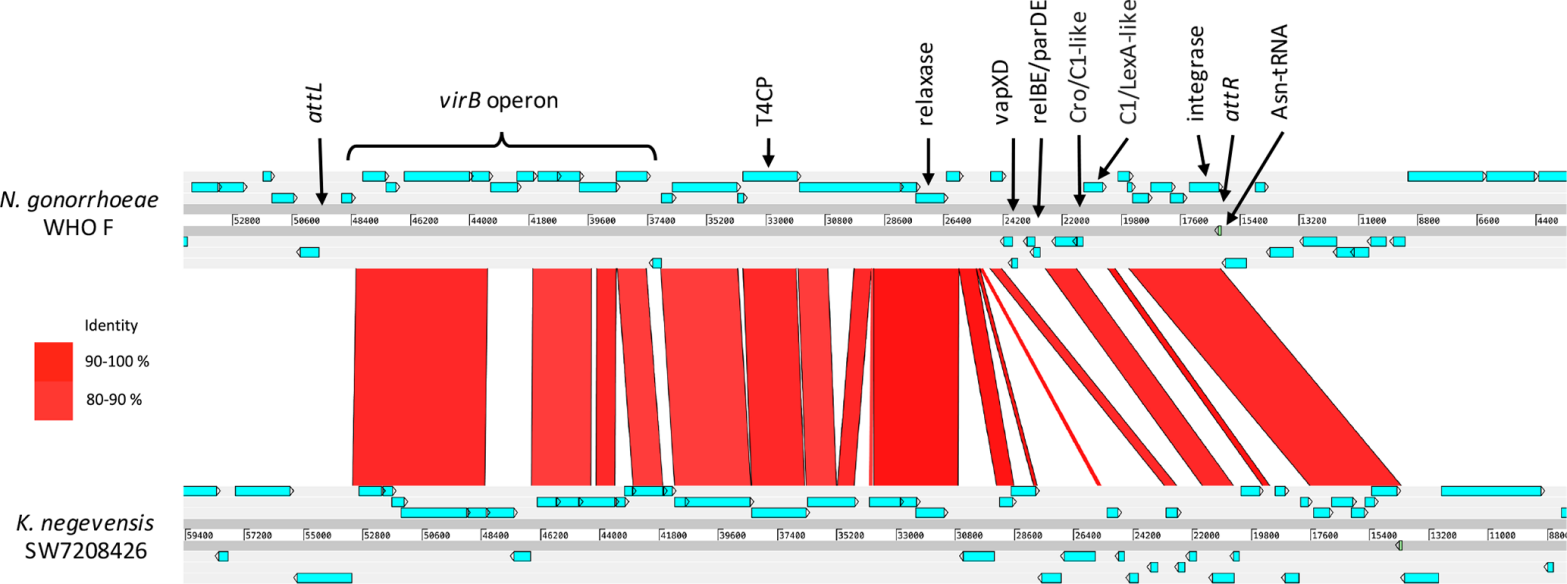
blastn comparisons of the *N. gonorrhoeae* WHO F ICE with its corresponding region in the possible donor species *K. negevensis *. High nucleotide identity and matching synteny imply shared evolutionary origins of this element. Core ICE genes (*virB4*, T4CP, relaxase and integrase) are highly conserved, but more variation exists between regions encoding TA systems, regulatory proteins and transposases. blast output was visualized using the Artemis Comparison Tool [35].

The most striking example of anDUS prevalence in a GI identified by our script was observed in the gonococcal reference strain WHO F [48], which possesses two almost identical copies of a 32 kb region each harbouring 20 AG-kingDUS (5ʹ-AGGCAGCCTGAA-3ʹ), 11 AG-simDUS (5ʹ-AGGCTGCCTGAA-3ʹ) and only 3 copies of the primary AT-DUS (Fig. 2b, islands indicated by turquoise blocks in the outer ring). This represents an approximately sevenfold reduction in prDUS 12-mer frequency relative to the chromosomal background and is accompanied by a local G+C-content reduction of 9.1 %. The GIs were delimited via comparison with the MS11 chromosome lacking these insertions, which revealed both insertion sites to be immediately downstream of asparagine tRNA genes. All AG-simDUS copies within these GIs form overlapping dyads with an AG-kingDUS, which was previously shown to be the most abundant DUS arrangement in *Simonsiella muelleri* [10], and which we found to be prevalent in commensals *Alysiella crassa* and *Kingella negevensis* (Table S1). Querying GI genes against NCBI databases (excluding gonococcal and meningococcal sequences) using blast consistently produced maximal identity hits from *K. negevensis* that were found to exhibit well-conserved synteny compared with their chromosomal arrangement in WHO F (Fig. 3) and were similarly located directly downstream of Asn-tRNA. Fig. 2e highlights this syntenic region within the *K. negevensis* genome, where it exhibits no atypical DUS or G+C-content characteristics.

The presence of a GI integrase gene adjacent to the tRNA insertion site (*attR*) in both species is consistent with canonical site-specific integrase-mediated integration, with approximately 75 % of integrases in Gram-negative hosts targeting tRNA or tmRNA genes as chromosomal integration sites [49]. Demarcation of the opposite GI boundary by a ~50 bp duplication of the Asn-tRNA 3ʹ end (*attL*) further supports this mechanism of integration. A search for essential genes of functional conjugation systems was carried out using CONJscan module of MacSyFinder [50, 51], which confirmed the presence of a mating-pair formation class T (MPF_T_) *virB* T4SS operon, MOB_P_ family relaxase and type IV coupling protein (T4CP)-encoding gene within the GI of both species (Fig. 3). Furthermore, a proteomics study [52] confirmed the expression of genes within the WHO F GI *virB* and relaxase region. The combination of a self-encoded conjugation system and integrase qualifies these GIs as ICEs, and since hosts of the closely related GIs are limited to *Kingella* spp., *N. gonorrhoeae *, *N. meningitidis* and *Neisseria lactamica* (discussed below), we will refer to this MGE family as ICE*riNK* (ICEs resident in *Neisseria* and *Kingella *). No pre-existing analogous ICE families were identified in a search of the ICEberg database [53] or The Transposon Registry [54], and no significant homology was observed from comparisons against gonococcal conjugative plasmids. Collectively, these results indicate extensive coevolution of ICE*riNK* with *K. negevensis* before a comparably recent transfer to *Neisseria* (most likely resulting from joint colonization of the human oropharynx [55]). Thus, we show the utility of DUS dialect plots for conveniently visualizing such intergeneric MGE acquisitions within *Neisseriaceae *.

Non-core ICE regions often carry a high degree of uncharacterized genes [56], but two TA loci in the relatively short ~6000 bp WHO F ICE*riNK* accessory region have defined homologues, although their precise biological functions remain to be determined [57–62]. The first of these belongs to the *relBE*/*ParDE* superfamily, which consists of structurally related *relE* mRNA interferases and *parE* DNA gyrase inhibitors. Collectively, members of this superfamily have putative roles in MGE stability, biofilm formation, and tolerance to oxidative stress and antibiotics [63–68]. The previously mentioned proteomic data set confirmed expression of this WHO F locus by detecting a product corresponding to the antitoxin gene [52]. The second TA system is a *vapXD* locus with ~95 % identity to its homologue located on the gonococcal cryptic plasmid: a plasmid carried by 96 % of isolates [69] but with no known associated phenotypic traits. Notably, WHO F was the only strain from the WHO 2016 reference genome panel not possessing a cryptic plasmid [48], suggesting any plasmid function may be substituted by chromosomally integrated *vapXD*. In addition to acquiring this usually plasmid-borne locus, gonococcal ICE*riNK* appears to inherit chromosomal genes based on the fact that ICE*riNK* sequences found in *N. meningitidis* carry loci otherwise specific to the *N. gonorrhoeae* chromosome (discussed below). Predicted products of two genes proximal to *attR* both contain a Cro/C1 helix-turn-helix DNA-binding domain, which is named after the Cro and C1 repressors that regulate the lytic–lysogenic transition of bacteriophage λ. The opposite orientation and shared upstream region of these ORFs is also consistent with the Cro/C1 system, as was observed for a pair of repressors involved in DNA-damage-induced activation of the SXT/R391 ICE family [70].

**Fig. 4.**
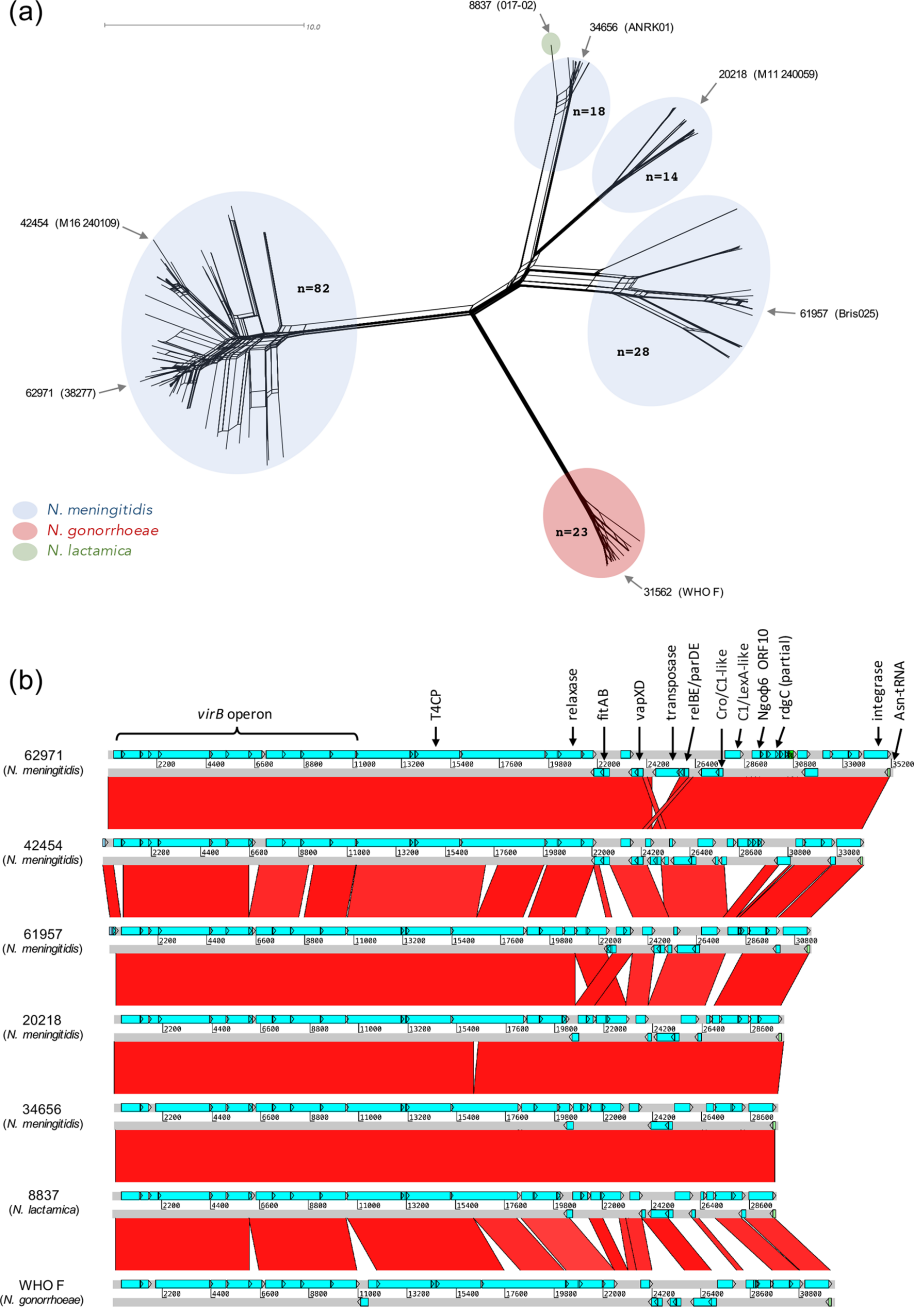
Visualizing variation in neisserial ICE*riNK* nucleotide sequences amongst PubMLST isolates. (a) Neighbour-Net network plotted with SplitsTree4 [42] using a distance matrix produced by comparison of allele values from the PubMLST VirB T4SS scheme, which includes many but not all ICE*riNK* genes. Each branch tip represents a single isolate and distances between tips correspond to the number of VirB T4SS loci with different allele sequences (commensurate with the scale bar). Coloured ellipses indicate sequence clusters and values in bold denote the number of isolates in each cluster. Distal arrows indicate the ID numbers and names of PubMLST isolate sequences selected as representatives of each cluster for further comparison in b. (b) Pairwise blastn comparisons of ICE*riNK* sequences from isolates listed in a. Connected regions have >85 % nucleotide identity. A detailed examination of ICE*riNK* variation across all isolates is beyond the scope of the present work, but comparison of this subset shows that the least conserved region lies between the relaxase and integrase genes.

**Fig. 5.**
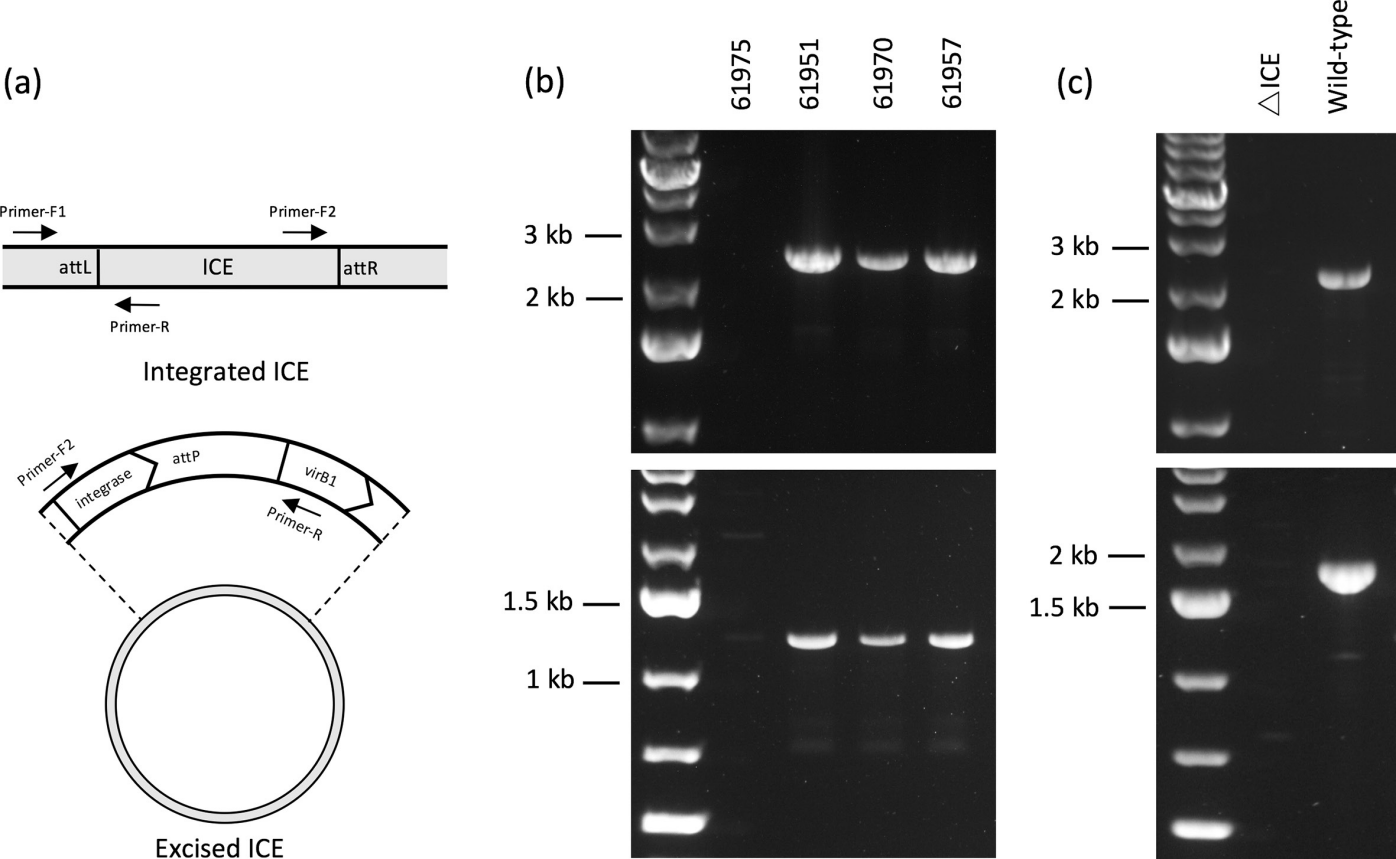
Using PCR amplification to confirm active excision of ICE*riNK* and ICE*Nmu* from their chromosomally integrated state to a circular form. (a) Schematic diagram depicting the primer orientations used in order to yield amplified products corresponding to chromosomally integrated ICEs (Primer-F1 and Primer-R) or the excised circular form (Primer-F2 and Primer-R). (b) Amplified products from meningococcal genomic DNA templates using primers NmeICE-F1 and NmeICE-R (upper panel) and plasmid DNA extract templates using primers NmeICE-F2 and NmeICE-R (lower panel). PubMLST genomic sequence data demonstrates the presence of ICE*riNK* in isolates 61951, 61970 and 61957, but the element is absent in 61 975. (c) Amplified products from *N. mucosa* ATCC 25996 genomic DNA templates with primers MucICE-F1 and MucICE-R (upper panel) and plasmid DNA extract templates with primers MucICE-F2 and MucICE-R (lower panel). A ΔICE strain was constructed by replacement of the entire ICE with a kanamycin-resistance cassette through transformation and homologous recombination.

To better characterize the abundance and sequence variation of this mobile element amongst clinical isolates, genomic sequences in the PubMLST *Neisseria* database were queried for the presence ICE*riNK* genes. This was expedited by utilizing the pre-existing VirB T4SS GI locus scheme that includes many core ICE*riNK* genes and provides each gene nucleotide variant with a unique allele number. This method does not capture all variation between ICE sequences of each isolate; however, it serves as a useful means to establish the presence or absence of the element and to identify more strongly conserved sequence clusters. A total of 180 isolates possessing core ICE*riNK* genes were detected, of which 14 were excluded due to ICE truncation at contig boundaries or isolate duplication, leaving 1 *N. lactamica *, 23 gonococcal and 142 meningococcal isolates: values that probably reflect the relative availability of PubMLST sequence data for each species, rather than representing a true bias in ICE host distribution. Comparisons of VirB T4SS scheme alleles for each isolate demonstrated that the ICE is highly conserved between all available gonococcal sequences, which form a single cluster, but varies significantly between meningococcal isolates, which are spread across four clusters (Fig. 4a, Table S2). Most variation between ICE*riNK* across all three species occurs between the relaxase and integrase genes and involves gain or loss of TA systems, phage-associated genes, transposases or uncharacterized ORFs, although several deletions/insertions are also present within the T4SS operon (Fig. 5b). The majority (58 %) of *N. meningitidis* ICE*riNK* sequences comprise a single cluster with several conserved features, the most striking of which are three homologues (>85 % amino acid sequence identity) of typically gonococcal-specific loci: *vapXD*, a 360 bp ORF of unknown function found in gonococcal filamentous prophages (NgoΦ6 ORF10) [71] and the *fitAB* chromosomal TA locus. FitAB is a VapBC family TA system hypothesized to contribute to gonococcal immune evasion and asymptomatic gonorrhoeal infection by reducing the rate of replication and trafficking within epithelial cells [72] and may, therefore, confer a similar regulated state of growth arrest to meningococcal strains possessing this ICE.

It is possible for an ICE to lose its excisive capability through genetic degradation and exist as a permanently integrated, solely vertically transmitted element [73, 74]. We sought to determine whether ICE*riNK* is present in both its chromosomally integrated and excised circular form; thus, indicating an active excision mechanism. This was achieved with three meningococcal isolates bearing ICE*riNK* (PubMLST ID numbers 61951, 61970 and 61957) via PCR amplification using primer pairs specific to either form of the element (Fig. 5a, b). Sequencing of amplicons from the circular ICE PCR revealed a typical ICE *att* site (*attP*) identical to a 60 bp chromosomal Asn-tRNA 3ʹ segment and terminating between regions encoding the D arm and anticodon arm stems (data not shown). Further investigation is required to establish the stability, modes of transfer and phenotypic consequences of this ICE family.

To the best of our knowledge, the only previously characterized neisserial ICE is a GGI-like meningococcal element, ICE*Nm*CC103, associated with an invasive clonal complex and suggested to contribute to persistence of this lineage by aiding meningococcal defences against oxidative stress-induced DNA damage [75]. blastn comparisons between ICE*Nm*CC103 and all ICE*riNK* sequences listed in Fig. 4 yielded no hits except for a single region present in isolates 62 971 and 42 454, which shares 94 % nucleotide identity to a C-terminal portion of the *rdgC* gene in ICE*Nm*CC103. A chromosomal homologue of *rdgC* is also present in meningococci and gonococci, where it is involved indirectly in antigenic variation of type IV pilus subunits [76, 77]. The disruption of *rdgC* in ICE*riNK* sequences may have resulted from direct insertion into the complete gene, as is the case for an ICE found in *Kingella kingae* [27].

### Characterization of a commensal *Neisseria* ICE

**Fig. 6.**
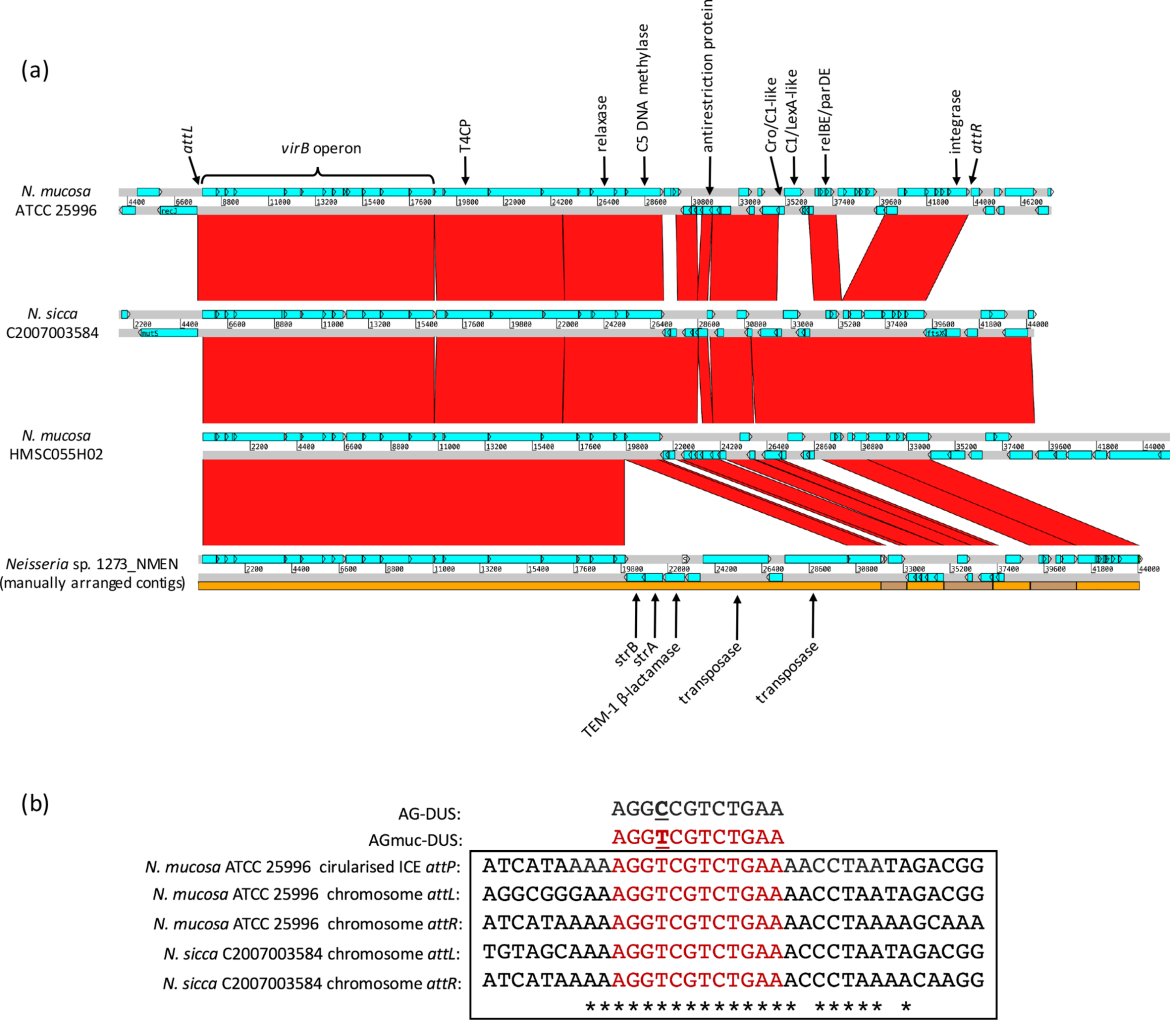
Comparisons of synteny and *att* sites of ICEs from *N. sicca* and *N. mucosa* genomes. (a) Pairwise blastn comparisons of ICEs highlighting the insertion of nested transposons encoding aminoglycoside phosphotransferases (*strA-strB*) and a β-lactamase in strain 1273_NMEN. The corresponding GenBank accession numbers are as follows: *N. mucosa* ATCC 25996 – GCA_000173875.1, *N. sicca* C2007003584 – GCA_003044425.1, *N. mucosa* HMSC055H02 – GCA_001813335.1, *Neisseria* sp. 1273_NMEN – GCA_001062585.1 (erroneously listed as *N. meningitidis* in NCBI databases). 1273_NMEN contigs were manually arranged by comparison with other ICEs (contigs are indicated by alternating orange and brown regions), but may not reflect true chromosomal adjacency. (b) Aligned *att* site sequences reveal 21 conserved bases containing a single copy of AG-mucDUS. *attP* of ATCC 25996 was determined by sequencing PCR products amplified from an extracted plasmid DNA template. Asterisks indicate fully conserved bases.

Screening of commensal *Neisseria* genomes for regions with under-represented prDUS and overrepresented anDUS also identified several GIs, the largest and most clearly delimited of which was a ~36 kb region in *N. mucosa* ATCC 25996 (Fig. 2f) possessing 38 copies of a single anDUS (AG-DUS), but 0 prDUS sites (AG-mucDUS; 5ʹ-AGGTCGTCTGAA-3ʹ). Notably, the G+C content of this GI (52.0 %) does not differ significantly from the genomic background (51.2 %), highlighting the ability of our method to detect islands that may be overlooked by traditional approaches. blast searches of NCBI databases with GI gene queries identified homologues with equivalent synteny in several genomes of closely related species (Fig. 6a), but no putative GI donor species with primary AG-DUS dialect could be identified. Similar to ICE*riNK*, these GIs are flanked by two short direct repeats (*attL* and *attR*), contain a complete MPF_T_ conjugation system with a MOB_P_ family relaxase and possess a terminal integrase gene, and can, therefore, be considered a group of ICEs (henceforth termed ICE*Nmu*). The ICE*Nmu* of strain ATCC 25996 was shown via PCR amplification to exist both in a chromosomally integrated and a circular form (Fig. 5c). However, significant differences were observed in the insertion sites, *attP* sequence and accessory genes of these ICEs compared with ICE*riNK*. Unlike common integrase-mediated integration, the chromosomal insertion sites of ICE*Nmu* are not within tRNA genes, instead being located between genes encoding a recombination/repair-associated protein (*recJ*) and hypothetical protein in *N. mucosa* ATCC 25995, and between a mismatch repair protein (*mutS*) and cell division protein (*ftsX*) gene for *N. sicca* C2007003584. Despite the different genomic locations of these two elements, their chromosomal *att* sites all share a conserved 21 bp motif whose presence in *attP* was demonstrated by sequencing of *N. mucosa* ATCC 25996 plasmid DNA with primers specific to the circularized ICE (Fig. 6b); thus, signifying integration via integrase-mediated site-specific recombination. Notably, *attP* contains an AG-mucDUS despite the predominance of the C→T transition variant AG-DUS throughout the rest of the element, suggesting a point mutation at this site – and possibly accompanying coevolution of the integrase – may have enabled host range expansion of ICE*Nmu* across uptake sequence dialect groups. The ability to integrate at highly abundant DUS sites would be a valuable adaptive trait for neisserial integrative MGEs due to greatly increasing the likelihood of successful integration following transfer. Although amino acid sequences for ICE*Nmu* and ICE*riNK* integrases are >95 % conserved within each family, comparisons between families show only ~55 % identity, which may underlie the differing insertion site preferences observed for each element. Additional GIs with ICE-like characteristics were detected in *Neisseria elongata *, *Neisseria iguanae* and *Neisseria zoodegmatis* (Fig. S2), but these occupied typical tRNA gene insertion sites and were not analysed further.

The majority of non-core ICE*Nmu* genes encode stability-related proteins including multiple TA systems, a C5 cytosine-specific methylase and an antirestriction protein, but *Neisserial* sp. 1273_NMEN bears an additional nested pair of transposons downstream of the *virB* operon that harbours a streptomycin-resistance gene pair (*strA-strB*) and TEM-1 β-lactamase, demonstrating the capacity for this ICE to act as a vehicle for modules conferring host fitness advantages.

## Discussion

The plasticity of neisserial genomes derives in a large part from intragenomic recombination, slipped-strand mispairing of tandem repeats and homologous recombination between close relatives with compatible DUS dialects [78, 79], but several studies have highlighted GIs whose atypical uptake sequence distribution and G+C content indicates acquisition from more phylogenetically distant species [29, 30, 80]. However, to the best of our knowledge, no previous studies have utilized overrepresentation of heterologous uptake sequence dialects in a screening strategy for GIs and their donor species in the ever-increasing body of bacterial genomic sequence data. The identification of two previously uncharacterized groups of ICEs demonstrates this method as a convenient way to highlight chromosomal MGE transfer within *Neisseriaceae *, but a similar method should also be applicable to members of *Pasteurellaceae *, which exhibit an analogous uptake sequence-mediated transformation specificity with subclade-specific sequence variants [81]. One caveat of any DUS-based approach to MGE identification is that it will likely only detect relatively recently acquired elements, as extensive coevolution tends to ‘ameliorate’ GI sequence characteristics to reflect that of the host genome [82]. However, the accumulation of uptake sequences can actually be exploited as a useful proxy for the degree of MGE-host coevolution, as was recently demonstrated for *Pasteurellaceae* prophages [83]. The deviation of other non-DUS nucleotide composition signatures, such as G+C content or codon usage, from their genomic baseline is commonly used to detect horizontally acquired regions [84]. However, intrageneric HGT events between relatives sharing similar compositional properties may be missed by this approach, or core genome regions misclassified as horizontally acquired [85, 86]. As shown here for ICE*Nmu*, our approach is able to identify GIs that would be undetectable by some traditional base composition methods.

Most ICE research to date has concentrated on a relatively small group of experimental model systems with accessory genes conferring selective phenotypes, such as resistance to antibiotics and heavy metals or metabolism of alternative nutrient sources [28]. This approach could lead to a narrow view of ICE biology by excluding those elements with more obscure functional payloads or with purely parasitic relationships with their host; thus, creating a need for phenotype-independent detection methods [56]. To this end, we have utilized the evolutionarily constrained genomic *k*-mer signatures provided by DUSs as a simple screening tool for GI ICE candidates, whose precise nature can be readily verified through homology-based search tools like blast or MacSyFinder [51]. Homologue searching of whole-genome data also provides an alternative MGE identification strategy to the method presented herein, but is clearly constrained by the choice of target proteins. This poses a particular problem for highly modular MGEs, for example, searching for conjugative elements via conserved T4SS components would not detect so-called ‘integrative mobilizable elements’, which mobilize *in trans* via conjugation systems encoded elsewhere in the genome [73]. Although we have focussed on ICEs in the current study, our DUS-based search approach benefits from a lack of restriction to specific MGE classes through the use of predefined homologues. The gene content of ICE*riNK* and ICE*Nmu,* consisting largely of TA systems and hypothetical proteins, does not clearly indicate whether the identified ICEs act as selfish genetic elements or could contribute to host fitness. TA systems were originally thought to function solely in MGE stabilization, but the discovery of widespread chromosomal TA loci prompted ongoing efforts to understand their additional biological roles [87]. One suggested role for certain TA systems in human colonizers is to increase tolerance to immune-mediated clearance or antibiotic treatment via growth arrest, which can manifest as latent or persistent infections [88–92]. Gonococcal *fitAB* is one such system, with a pronounced inhibitory effect on growth and traversal rates within epithelial cells [93]. The presence of *fitAB* in the majority of *N. meningitidis* ICE*riNK* sequences indicates that ICE TA systems may have functional roles beyond maintaining element stability, but further investigation is required to establish whether the typical gonococcal *fitAB*-dependent reduction in intracellular growth and trafficking is retained by meningococci possessing this element.

Six potential global proteomic markers for antimicrobial resistance in *N. gonorrhoeae* have been identified based on their differential expression in the fully antibiotic susceptible WHO F compared to 14 strains with varied resistance profiles [52]; however, genes corresponding to three of these proteins (WHO_F_01139, WHO_F_01144 c and WHO_F_01126) are located within chromosomal ICE*riNK* regions. Since ICE*riNK* is absent in the other strains analysed, differential expression is likely to result from the incidental presence of this MGE rather than a causal link with antimicrobial susceptibility, which highlights the importance of identifying ICEs regardless of their direct clinical impact. An in-depth analysis of the sequence variation between ICE*riNK* variants and their association with other genomic features is beyond the scope of the present work but, given the number and diversity of available sequences, should provide insights into ICE host adaptation.

ICE*Nmu* is the first example of a MGE family seemingly able to exploit DUS sites as insertional hotspots, which could reflect an evolutionary strategy facilitating host range broadening across DUS dialect groups via point mutations in *attP*. In addition to ensuring numerous possible integration sites in a recipient genome, *attP* DUS sites may increase ICE transfer rates during expansion to new host groups by enabling uptake via DUS-specific natural transformation to complement or substitute conjugation. Although integration of ICE*Nmu* via its self-encoded integrase remains to be experimentally verified, an integrase preference for DUS sequences would be consistent with typical recombinase biases towards symmetrical integration sites [49], because DUSs frequently occur in inverted repeat pairs suggested to function as transcriptional terminators [6, 94]. The positioning of these DUS pairs at putative transcription stop points would ensure that MGEs utilizing them as integration sites have a reduced chance of causing deleterious mutations compared with those interrupting upstream regulatory sequences or intragenic regions. However, both insertion sites for ICE*NMu* identified here are located upstream of genes involved in DNA repair, for which altered expression could increase ICE stability by preventing element removal via repair-mediated ‘chromosomal curing’ [95].

In conclusion, we have validated the use of prDUS motif under-representation combined with anDUS dialect overrepresentation to detect mobile GIs within *Neisseriaceae *. Two GI groups were identified by their abundance of atypical DUS sites and shown to be excisable ICEs with *attP* sequences corresponding to different chromosomal insertion sites: an Asn-tRNA gene for ICE*riNK* and a non-canonical intergenic 21 bp motif centred around a prDUS for ICE*Nmu*. These newly characterized elements highlight the impressive ability of ICEs to flourish in diverse host species, in some cases by co-opting pre-existing adaptive characteristics of the host. Future investigation of ICE*riNK* should address whether sequence clusters correlate with other genomic features and specific disease or carriage characteristics, as well as determining the role of *fitAB* in the major meningococcal sequence cluster. Functional analyses of ICE*Nmu* should clarify the role of the self-encoded integrase in DUS site integration and establish whether the *attP* DUS site influences transfer frequencies via DUS-specific uptake.

## Data Bibliography

1. Abrams AJ, Trees DL, Nicholas RA, GenBank accession no. GCA_001047225.1, *N. gonorrhoeae* FA19 (2015).

2. Mitreva M, Pepin KH, Mihindukulasuriya KA, Fulton R, Fronick C, O'Laughlin M, Miner T, Herter B, Rosa BA, Cordes M, Tomlinson C, Wollam A, Palsikar VB, Mardis ER, Wilson RK, GenBank accession no. GCA_001836705.1 and GCA_001815375.1, Neisseria sp. HMSC061B04 and HMSC065C04 (2016).

3. Ward D, Seifert HS, Walker B, Young SK, Zeng Q, Gargeya S, Fitzgerald M, Haas B, Abouelleil A, Alvarado L, Arachchi HM, Berlin AM, Chapman SB, Goldberg J, Griggs A, Gujja S, Hansen M, Howarth C, Imamovic A, Larimer J, McCowan C, Montmayeur A, Neiman D, Pearson M, Priest M, Roberts A, Saif S, Shea T, Sisk P, Sykes S, Wortman J, Nusbaum C, Birren B, GenBank accession no. GCA_000156855.2, *N. gonorrhoeae* MS11 (2013).

4. Unemo M, Golparian D, Sánchez-Busó L, Grad Y, Jacobsson S, Ohnishi M, Lahra MM, Limnios A, Sikora AE, Wi T, Harris SR, GenBank accession no. GCA_900087635.2, *N. gonorrhoeae* WHO F (2016).

5. Tettelin H, Saunders NJ, Heidelberg J, Jeffries AC, Nelson KE, Eisen JA, Ketchum KA, Hood DW, Ciecko A, Peden JF, Dodson RJ, Nelson WC, Gwinn ML, Peterson JD, Hickey EK, Haft DH, Salzberg SL, White O, Fleischmann RD, Dougherty BA, Mason TM, Parksey DS, Blair E, Cittone H, Clark EB, Cotton MD, Utterback TR, Khouri HM, Qin H, Vamathevan J, Gill J, Scarlato V, Masignani V, DeBoy RT, Pizza M, Grandi G, Sun L, Smith HO, Fraser CM, Moxon ER, Rappuoli R, Venter JC, GenBank accession no. GCA_000008805.1, *N. meningitidis* MC58 (2005).

6. Krauland MG, Dunning Hotopp JC, Riley DR, Parankush Das S, Nadendla S, Daugherty SC, Tallon LJ, Sadzewicz L, Marsh JW, Fraser CM, Harrison LH, GenBank accession no. GCA_000328265.2, *N. meningitidis* NM442 (2012).

7. Earl A, Ward D, Feldgarden M, Gevers D, Izard J, Baranova OV, Blanton JM, Tanner AC, Dewhirst F, Young SK, Zeng Q, Gargeya S, Fitzgerald M, Haas B, Abouelleil A, Alvarado L, Arachchi HM, Berlin A, Brown A, Chapman SB, Chen Z, Dunbar C, Freedman E, Gearin G, Goldberg J, Griggs A, Gujja S, Heiman D, Howarth C, Larson L, Lui A, MacDonald PJP, Montmayeur A, Murphy C, Neiman D, Pearson M, Priest M, Roberts A, Saif S, Shea T, Shenoy N, Sisk P, Stolte C, Sykes S, Wortman J, Nusbaum C, Birren B, GenBank accession no. GCA_000163775.2, *S. muelleri* ATCC 29453 (2012).

8. Kyrpides N, Huntemann M, Han J, Chen A, Mavromatis K, Markowitz V, Palaniappan K, Ivanova N, Schaumberg A, Pati A, Liolios K, Nordberg HP, Cantor MN, Hua SX, Woyke T, GenBank accession no. GCA_000745955.1, *A.crassa* DSM 2578 (2014).

9. El Houmami N, Bakour S, Bzdrenga J, Rathored J, Seligmann H, Robert C, Armstrong N, Schrenzel J, Raoult D, Yagupsky P, Fournier PE, GenBank accession no. GCA_900177895.1, *K. negevensis* SW7208426 (2017).

10. Hughes-Games A, Figshare, DOI: 10.6084/m9.figshare.11542011, spreadsheet of references and metadata for all PubMLST isolates analysed (2020).

11. Fulton L, Clifton S, Chinwalla AT, Mitreva M, Sodergren E, Weinstock G, Clifton S, Dooling DJ, Fulton B, Minx P, Pepin KH, Johnson M, Bhonagiri V, Nash WE, Mardis ER, Wilson RK, GenBank accession no. GCA_000173875.1, GenBank accession no. GCA_900177895.1, *N. mucosa* ATCC 25996 (2009).

12. Nichols M, Topaz N, Wang X, Wang X, Boxrud D, GenBank accession no. GCA_003044425.1, *N. sicca* C2007003584 (2018).

13. Mitreva M, Pepin KH, Mihindukulasuriya KA, Fulton R, Fronick C, O'Laughlin M, Miner T, Herter B, Rosa BA, Cordes M, Tomlinson C, Wollam A, Palsikar VB, Mardis ER, Wilson RK, GenBank accession no. GCA_001813335.1, *Neisseria* sp. HMSC055H02 (2016).

14. Roach DJ, Burton JN, Lee C, Stackhouse B, Butler-Wu SM, Cookson BT, Shendure J, Salipante SJ, GenBank accession no. GCA_001062585.1 *Neisseria* sp. 1273_NMEN (originally classified as *N. meningitidis* in error) (2015).

15. Topaz N, Boxrud D, Retchless AC, Nichols M, Chang H-Y, Hu F, Wang X, GenBank accession no. GCA_003351685.1, *N. elongata* M15910 (2018).

16. Gui Z, GenBank accession no. GCA_003013245.1, *N. iguanae* ATCC 51483 (2018).

17. Pathogen Informatics (Sanger), GenBank accession no. GCA_900187305.1, *N. zoodegmatis* NCTC12230 (2017).

## Supplementary Data

Supplementary material 1
