# Supplementary material for: Identification of integrative and conjugative elements in pathogenic and commensal Neisseriaceae species via genomic distributions of DNA uptake sequence dialects
Source: Microb Genom. 2020 May 4;6(5):e000372. doi: 10.1099/mgen.0.000372 (PMC7371117; doi:10.1099/mgen.0.000372)
Supplement: Supplementary material 1 [file mgen-6-372-s001.pdf]

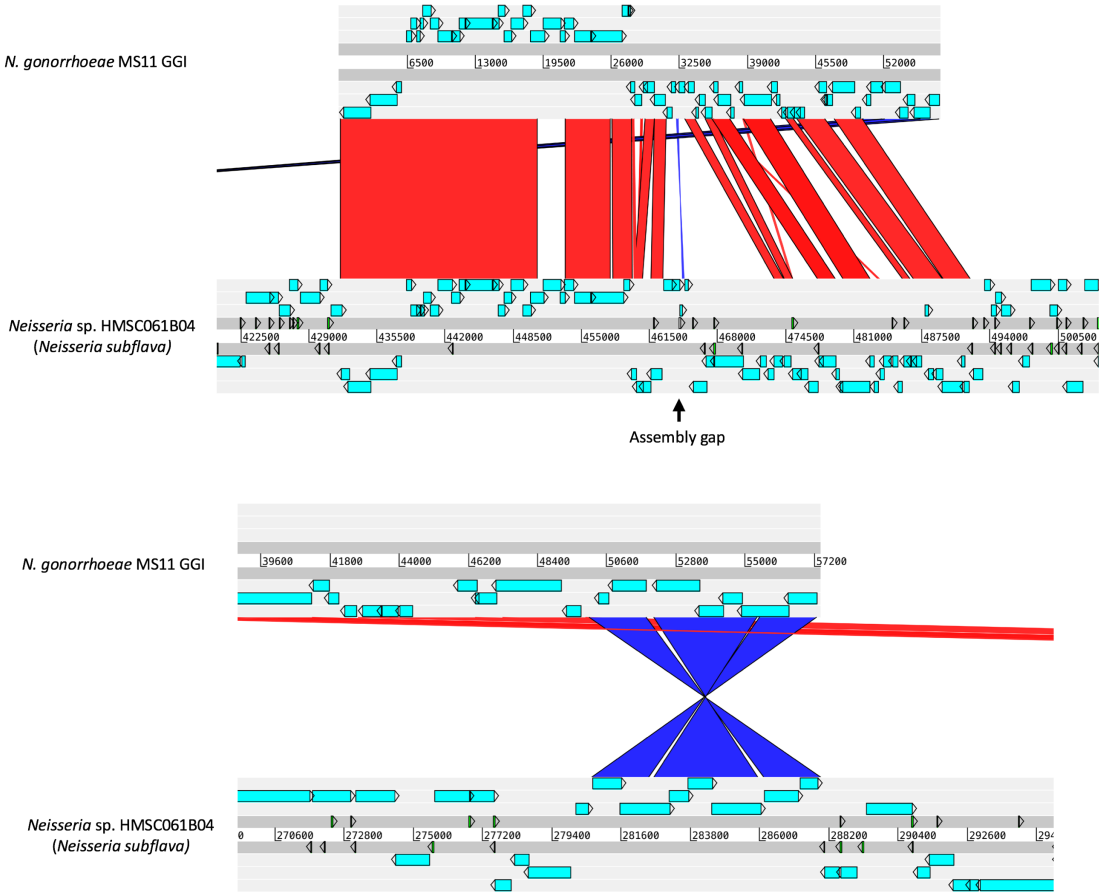

Figure S1. Pairwise BLAST comparisons of the gonococcal genomic island with genomic regions from *Neisseria subflava* LTQL00000000. Connected regions have 88-96% identity. The two regions of LTQL00000000 with homology to the GGI shown here are both located at contig ends, suggesting their separation may be an assembly artefact. The high identity and matching syntenies indicate *N. subflava* as a likely donor of this genomic island to *N. gonorrhoeae*.

id	isolate	country	year	species	NEIS2323 (virB1)	NEIS2324 (virB2)	NEIS2325 (virB3)	NEIS2326 (virB4)	NEIS2327 (virB5)	NEIS2328 (virB6)	NEIS2329 (virB7)	NEIS2330 (virB8)	NEIS2331 (virB9)	NEIS2332 (virB10)	NEIS2333 (virB11)	NEIS2334 (topA/topB)	NEIS2335 (T4CP/virBD4/traG)	NEIS2336	NEIS2337	NEIS2338 (relaxase/virBD2)	NEIS2339	NEIS2340 (fitB/stbB)	NEIS2341 (fitA/stbC)	NEIS2342	NEIS2343	NEIS2344	NEIS2345 (Ngoφ6 ORF10)	NEIS2347	NEIS2348	NEIS2349	NEIS2350 (trbM)	NEIS2351 (trbL)	NEIS2352 (traM)	NEIS2353	NEIS2354 (integrase)
26040	93-N213	Australia	1993	*Neisseria meningitidis*	1	1	1	1	1	1	1	1	1	1	1	1	1	1	1	1	1	1	1	1	1	1	1	1	1	1	1	1	1	1	1
37759	M14 240623	UK	2014	*Neisseria meningitidis*	1	1	1	3	9	1	1	1	1	6	2	1	5	6	1	14	1	2	1	1	1	1		1	3	4	1	1	3	1	9
42139	IPM32	Morocco	2012	*Neisseria meningitidis*	1	1	1	3	7	2		1	1	3	14	1	5	23	1	1	1	2	1	1	1	1	1	1	3	1	1	1	3	1	1
43834	IPM134	Morocco	2015	*Neisseria meningitidis*	1	1	1	3	7		1	1	1	6	16	1	5	24	8	1	1	2	1	1	1	1	24		3	1	1	1	3	1	1
43843	IPM143	Morocco	2015	*Neisseria meningitidis*	1	1	1	3	7	2		1	1	3	2	1	5	6	1	17		2	1	1	1	1	21		3	1	1	1	3	1	1
47065	PT4	Italy	2016	*Neisseria meningitidis*	1	1	1	1	1	1	1	15	1	1	1	1	19	1	1	1	1	1	1	1	1	1	1	1	1	1	1	1	1	1	1
47156	PT99	Italy	2016	*Neisseria meningitidis*	1	1	1	29	11		3	1	1	24	2	1	5	29	1	1	1	62	1	1	22	1	1	1	3	1	1		3	1	12
50656	M38901	USA	2015	*Neisseria meningitidis*	1	1	1	24	7	1	1	1	1	6	2	1	5	7	1	1	11	2	1	1	1	1	1	1	3	1	1	1	3	1	1
51318	R056	UK	2015	*Neisseria meningitidis*	1	1	1	33	11		1	1	1	6	2	1	5	36	1	1	1	62	1	1	1	1	1	1	3	1	1	1	3	1	12
54628	LNP24347abd	France	2007	*Neisseria meningitidis*	1	1	1	3	7	2		1	1	3	14	1	5	6	1	1	1	2	1	1	1	1	1	1	3	1	1	1	3	1	1
56718	NLMB069	Canada	2014	*Neisseria meningitidis*	1	1	1	3		5		1	1	6	2	1	5		1	1	1		1	1	1	1	4				1	1	3	1	1
57248	NMLB069	Canada	2014	*Neisseria meningitidis*	1	1	1	3		5		1	1	6	2	1	5		1	1	1		1	1	1	1	4				1	1	3	1	1
59075	SMG_18_405	UK	2018	*Neisseria meningitidis*	1	1	1	29	11		3	1	1	24	2	1	5	29	11	1	1	62	1	1	22	1	1	1	3	1	1	1	3	1	12
59854	C10695	South Africa	2017	*Neisseria meningitidis*	1	1	1	3	7		1	1	1	6	16	1	5	24	1	1	1	2	1	1	1	1	1	1	3	1	1	1	3	1	1
340	196/87	Norway	1987	*Neisseria meningitidis*	3	1	1	3	2	2	1	1	1	3	2	1	2	3	1	1	1	2	1	1	1	1	1	1	3	1	1	1	3	1	1
39854	M05749	Norway	1987	*Neisseria meningitidis*	3	1	1	3	2	2	1	1	1	3	2	1	2	3	1	1	1	2	1	1	1	1	1	1	3	1	1	1	3	1	1
40450	LNP27654	France	2014	*Neisseria meningitidis*	13	1	1	3	7	2	1	1	1	3	2	1	5	6	1	1	1	2	1	1	1	1	21		3	1	1	1	3	1	1
30308	NM8307	UK	2011	*Neisseria meningitidis*	1	1	1	3	7	1	1	1	1	6	2	5	5	6	3	1	1	2	1	1	1	1	1	1	3	1	1	1	3	1	1
30320	NM8736	UK	2011	*Neisseria meningitidis*	1	1	1	3	7	1	1	1	1	6	2	5	5	6	1	1	1	2	1	1	1	1	1	1	3	1	1	1	3	1	1
30335	NM10492	UK	2013	*Neisseria meningitidis*	1	1	1	9	7	1	1	1	1	6	2	5	5	9	1	1	1	2	1	1	1	1	1	1	3	2	1	1	3	1	1
31177	NM8307	UK	2011	*Neisseria meningitidis*	1	1	1	3	7	1	1	1	1	6	2	5	5	6	3	1	1	2	1	1	1	1	1	1	3	1	1	1	3	1	1
31189	NM8736	UK	2011	*Neisseria meningitidis*	1	1	1	3	7	1	1	1	1	6	2	5	5	6	1	1	1	2	1	1	1	1	1	1	3	1	1	1	3	1	1
31204	NM10492	UK	2013	*Neisseria meningitidis*	1	1	1	9	7	1	1	1	1	6	2	5	5	9	1	1	1	2	1	1	1	1	1	1	3	2	1	1	3	1	1
36133	12006_2015	Ireland	2015	*Neisseria meningitidis*	1	1	1	3	7	1	1	1	1	6	2	5	5	6	1	1	1	2	1	1	1	1	1	1	3	1	1	1	3	1	1
36815	12006_2015b	Ireland	2015	*Neisseria meningitidis*	1	1	1	3	7	1	1	1	1	6	2	5	5	6	1	1	1	2	1	1	1	1	1	1	3	1	1	1	3	1	1
47079	PT18	Italy	2016	*Neisseria meningitidis*	1	1	1	28	7	1	1	1	1	6	2	5	5	31	1	1	1	2	1	1	1	1	1	1	3	1	1	1	3	1	1
50611	M38608	USA	2015	*Neisseria meningitidis*	1	1	1	3	7	1	1	1	1	6	2	5	5	6	1	1	1	2	1	1	1	1	1	1	3	1	1	1	3	1	1
55144	2162152	The Netherlands	2016	*Neisseria meningitidis*	1	1	1	3	7	1	1	1	1	6	2	5	5	6	1	1	1	2	1	1	1	1	1	1	3	1	1	1	3	1	1
56121	NM03051	USA	2007	*Neisseria meningitidis*	1	1	1	3	7	1	1	1	1	6	2	5	5	6	1	1	1	2	1	1	1	1	1	1	3	1	1	1	3	1	1
58178	SMG_18_68	UK	2017	*Neisseria meningitidis*	1	1	1	3	7	1	1	1	1	6	2	5	5	6	1	1	1	2	1	1	1	1	1	1	3	1	1	1	3	1	1
59322	NM03056	USA	2007	*Neisseria meningitidis*	1	1	1	3	7	1	1	1	1	6	2	5	5	6	1	1	1	2	1	1	1	1	1	1	3	1	1	1	3	1	1
59418	NM03095	USA	2007	*Neisseria meningitidis*	1	1	1	3	7	1	1	1	1	6	2	5	5	6	1	1	1	2	1	1	1	1	1	1	3	1	1	1	3	1	1
61161	NmissMEN3	Italy	2016	*Neisseria meningitidis*	1	1	1	3	7	1	1	1	1	6	2	5	5	6	1	1	1	2	1	1	1	1	1	1	3	1	1	1	3	1	1
61162	NmissMen5	Italy	2016	*Neisseria meningitidis*	1	1	1	3	7	1	1	1	1	6	2	5	5	6	1	1	1	2	1	1	1	1	1	1	3	1	1	1	3	1	1
61163	NmissMEN6	Italy	2016	*Neisseria meningitidis*	1	1	1	3	7	1	1	1	1	6	2	5	5	6	1	1	1	2	1	1	1	1	1	1	3	1	1	1	3	1	1
61170	NmissMEN13	Italy	2016	*Neisseria meningitidis*	1	1	1	3	7	1	1	1	1	6	2	5	5	6	1	1	1	2	1	1	29	1	1	1	8	1	1	1	3	1	1
61171	NmissMEN14	Italy	2016	*Neisseria meningitidis*	1	1	1	3	7	1	1	1	1	6	2	5	5	6	1	1	1	2	1	1	1	1	1	1	3	1	1	1	3	1	1
61172	NmissMEN15	Italy	2016	*Neisseria meningitidis*	1	1	1	3	7	1	1	1	1	6	2	5	5	6	1	1	1	2	1	1	1	1	1	1	3	1	1	1	3	1	1
61186	NmissMEN58	Italy	2016	*Neisseria meningitidis*	1	1	1	3	7	1	1	1	1	6	2	5	29	8	1	1	1	2	1	1	30	1	1	1	3	1	1	1	3	1	1
61188	NmissMEN61	Italy	2016	*Neisseria meningitidis*	1	1	1	3	7	1	1	1	1	6	2	5	5	6	1	1	1	2	1	1	1	1	1	1	3	1	1	1	3	1	1
62971	38277	Unknown	2014	*Neisseria meningitidis*	1	1	1	3	7	1	1	1	1	6	2	5	5	44	1	1	1	2	1	1	1	1	1	1	3	1	1	1	3	1	1
63034	NmissMEN4	Italy	2016	*Neisseria meningitidis*	1	1	1	3	7	1	1	1	1	6	2	5	5	6	1	1	1	2	1	1	31	1	1	1	3	1	1	1	3	1	1
63036	NmissMEN35	Italy	2016	*Neisseria meningitidis*	1	1	1	3	7	1	1	1	1	6	2	5	5	6	1	1	1	2	1	1	1	1	1	1	3	1	1	1	3	1	1
83773	MCbar-778	Sweden		*Neisseria meningitidis*	11	1	1	3	7	1	1	1	1	6	2	5	5	6	1	1	1	2	1	1	1	1	1	1	3	1	1	1	3	1	1
30318	NM8674	UK	2011	*Neisseria meningitidis*	1	1	1	8		5		4	3	6	2	7	5	7	1	1	1	2	1	1	1	1		1	3	1	1	1	3	1	1
31187	NM8674	UK	2011	*Neisseria meningitidis*	1	1	1	8		5		4	3	6	2	7	5	7	1	1	1	2	1	1	1	1	1	1	3	1	1	1	3	1	1
42645	15-8703451	UK	2015	*Neisseria meningitidis*	1	1	1	8		5		4	3	6	2	7	5	7	1	1	1	2	1	1	1	1	23	1	3	1	1	1	3	1	1
50460	NML161	Canada	2015	*Neisseria meningitidis*	1	1	1	8		5		4	3	6	2	7	5	7	1	1	1	2	1	1	1	1	1	1	3	1	1	1	3	1	1
53427	107553	Finland	2017	*Neisseria meningitidis*	1	1	1	8		5		4	3	6	2	7	26	7	1	1	1	2	1	1	1	1	1	1	3	1	1	1	3	1	1
54019	M37064	Brazil	2014	*Neisseria meningitidis*	1	1	1	8		5		4	3	6	2	7	5	39	1	1	1	2	1	1	1	1	1	1	3	1	1	1	3	1	1
54059	M37153	Brazil	2014	*Neisseria meningitidis*	1	1	1	8		5		4	3	6	22	7	5	7	9	1	1	2	1	1	1	1	1	1	3	1	1	1	3	1	1
61164	NmissMEN7	Italy	2016	*Neisseria meningitidis*	1	1	1	8		5		4	3	6	2	7	5	7	1	1	1	2	1	1	1	1	1	1	3	1	1	1	3	1	1
61169	NmissMEN12	Italy	2016	*Neisseria meningitidis*	1	1	1	8		5		4	3	6	2	7	5	7	1	1	1	2	1	1	1	1	1	1	3	1	1	1	3	1	1
61174	NmissMEN18	Italy	2016	*Neisseria meningitidis*	1	1	1	8		5		4	3	6	2	7	5	7	1	1	1	2	1	1	1	1	1	1	3	1	1	1	3	1	1
61178	NmissMEN39	Italy	2016	*Neisseria meningitidis*	1	1	1	8		5		4	3	6	2	7	5	7	1	1	1	2	1	1	1	1	1	1	3	1	1	1	3	1	1
61182	NmissMEN53	Italy	2016	*Neisseria meningitidis*	1	1	1	8		5		4	3	6	2	7	5	7	1	1	1	2	1	1	1	1	1	1	3	1	1	1	3	1	1
63038	NmissMEN52	Italy	2016	*Neisseria meningitidis*	1	1	1	8		5		4	3	6	2	7	5	7	1	1	1	2	1	1	1	1	1	1	3	1	1	1	3	1	1
42454	M16 240109	UK	2016	*Neisseria meningitidis*	12	1	1	8		5		4	3	6	2	7	5	7	1	1	1	2	1	1	16	1	1	1	3	6	1	1	3	6	1
53094	M16 240759	UK	2016	*Neisseria meningitidis*	18	1	1	36		15		4	3	6	2	7	5	7	1	1	1	2	1	1	1	1	1	1	3	1	1	1	3	1	1
63040	NmissMEN59	Italy	2016	*Neisseria meningitidis*	19	1	1	8		5		4	3	6	2	7	5	7	9	1	1	2	1	1	1	1	1	1	3	1	1	1	3	1	1
30323	NM9124	UK	2012	*Neisseria meningitidis*	1	1	1	3	7	1	1	1	1	6	2	8	5	8	1	1	1	2	1	1	1	1	1	1	3	1	1	1	3	1	1
30337	NM10763	UK	2013	*Neisseria meningitidis*	1	1	1	3	7	1	1	5	1	6	2	8	5	10	1	1	1	2	1	1	1	1	1	1	3	1	1	1	3	1	1
31192	NM9124	UK	2012	*Neisseria meningitidis*	1	1	1	3	7	1	1	1	1	6	2	8	5	8	1	1	1	2	1	1	1	1	1	1	3	1	1	1	3	1	1
31206	NM10763	UK	2013	*Neisseria meningitidis*	1	1	1	3	7	1	1	5	1	6	2	8	5	10	1	1	1	2	1	1	1	1	1	1	3	1	1	1	3	1	1
38879	P14	Italy	2012	*Neisseria meningitidis*	1	1	1	3	7	1	1	1	1	6	2	8	5	8	1	1	1	2	1	1	1	1	1	1	3	1	1	1	3	1	1
47082	PT21	Italy	2016	*Neisseria meningitidis*	1	1	1	3	7	1	1	1	1	6	2	8	5	8	1	1	1	2	1	1	1	1	1	1	3	1	1	1	3	1	1
48404	SRR3357141	UK	2014	*Neisseria meningitidis*	1	1	1	3	7	1	1	1	1	6	2	8	5	8	1	1	1	2	1	1	1	1	1	1	3	1	1	1	3	1	1
53248	M17 240048	UK	2017	*Neisseria meningitidis*	1	1	1	3	7	1	1	1	1	6	2	8	5	8	1	1	1	2	1	1	1	1	1	1	3	1	1	1	3	1	1
61183	NmissMEN54	Italy	2016	*Neisseria meningitidis*	1	1	1	3	7	1	1	1	1	6	2	8	5	8	1	1	1	2	1	1	1	1	1	1	3	1	1	1	3	1	1
61187	NmissMEN60	Italy	2016	*Neisseria meningitidis*	1	1	1	3	7	1	1	1	1	6	2	8	5	8	9	1	1	2	1	1	1	1	1	1	3	1	1	1	3	1	1
63033	NmissMEN1	Italy	2016	*Neisseria meningitidis*	1	1	1	3	7	1	1	1	1	6	2	8	5	8	1	1	1	2	1	1	1	1	1	1	3	1	1	1	3	1	1
21146	M11 240597	UK	2011	*Neisseria meningitidis*	1	1	1	3		5	1	1	1	12	2	15	5	6	1	1	1	2	1	3	1	1	1	1	3	1	1	1	3	5	1
51857	W-5959	Greece	2016	*Neisseria meningitidis*	1	1	1	3		5	1	1	1	12	2	15	24	6	1	1	1	2	1	3	1	1	1	1	3	7	1	1	3	5	1
51860	W-5981	Greece	2016	*Neisseria meningitidis*	1	1	1	3		5	1	1	1	12	2	15	5	6	1	1	1	2	1	3	1	1			3	7	1	1	3	5	1
38178	M37959	Brazil	2014	*Neisseria meningitidis*	1	1	8	8		10	1	12	3	6	2	21	5	22	1	1	1	2	1	1	1	1	1	1	3	1	1	1	3	1	1
54062	M37959	Brazil	2014	*Neisseria meningitidis*	1	1	8	8		10	1	12	3	6	2	21	5	22	1	1	1	2	1	1	1	1	1	1	3	1	1	1	3	1	1
39456	M08 240169	UK	2008	*Neisseria meningitidis*	1	1	1	24	7	1	1	1	1	6	2	22	5	7	1	1	1	2	1	1	1	1	1	1	3	1	1	1	3	1	1
50729	M40216	USA	2016	*Neisseria meningitidis*	1	1	1	3	7	1	1	1	1	6	2	27	5	6	1	1	1	2	1	1	1	1	1	1	3	1	1	1	3	1	1
54576	LNP16397abd	France	1998	*Neisseria meningitidis*	1	1	1	3	11		3	1	1	6	2	29	5	29	1	1	12	62	1	1	27	1	21		3	1	1	1	3	1	12
58844	SMG_18_252	UK	2018	*Neisseria meningitidis*	1	1	1	3	7	1	1	1	1	6	2	5	5	8	1	1	1	2	1	1			1	1	3	1	1	1	3	1	1
61173	NmissMEN16	Italy	2016	*Neisseria meningitidis*	1	1	1	3	7	1	1	1	1	6	2	5	5	8	1	1	1	71	1	1			1	1	3	1	1	1	3	1	1
55162	2171800	The Netherlands	2017	*Neisseria meningitidis*	1	1	1	3	7	1	1	1	1	6	2	31	5	6	1	1	1	2	1	1	1	1	1	1	3	1	1	1	3	1	1
26024	58	Japan	1979	*Neisseria meningitidis*	4	2	3	7	4	4		3	2	4	4	3	4	4	2	2	2			2	3	2					2	2	4	3	3
27810	M12 240694	UK	2012	*Neisseria meningitidis*	4	2	2	5	4	4		3	2	4	4	3	4	4	2	2	2			2	3	2			4		2	2	4	3	3
30045	M01 242572	UK	2001	*Neisseria meningitidis*	4	2	2	5	4	4		3	2	4	4	3	4	4	2	2	2			2	3	2			4		2	2	4	3	3
34647	NM3173	USA	2009	*Neisseria meningitidis*	4	2	2	5	4	4		3	2	4	4	3	4	4	2	2	2			2	3	2			4		2	2	4	3	3
42651	15-8708206	UK	2015	*Neisseria meningitidis*	4	2	2	5	4	4		3	2	4	4	3	4	4	2	2	2			2	3	2			4		2	2	4	3	3
44876	06R0129091	Iceland	2006	*Neisseria meningitidis*	4	2	10	5	4	4		3	2	4	4	3	4	4	2	2	2			2	3	2			4		2	2	4	3	3
44879	0610120109	Iceland	2006	*Neisseria meningitidis*	4	2	10	5	4	4		3	2	4	4	3	4	4	2	2	2			2	3	2			4		2	2	4	3	3
51903	17-36	Sweden	2017	*Neisseria meningitidis*	4	2	2	34	4	4		3	2	4	4	3	4	4	2	2	2			2	3	2			4		2	2	4	3	3
59210	NM02607	USA	2007	*Neisseria meningitidis*	4	2	2	5	4	4		3	2	4	4	3	4	4	2	2	2			2	3	2			4		2	2	4	3	3
61951	Bris006	UK	2014	*Neisseria meningitidis*	4	2	2	5	4	4		3	2	19	4	3	4	4	2	16	10			2	3	2			4		2	2	4	3	3
61957	Bris025	UK	2014	*Neisseria meningitidis*	4	2	2	5	4	4		3	2	19	4	3	4	4	2	16	10			2	3	2			4		2	2	4	3	3
61970	Bris008	UK	2014	*Neisseria meningitidis*	4	2	2	5	4	4		3	2	19	4	3	4	4	2	16	10			2	3	2			4		2	2	4	3	3
83769	MCbar-671	Sweden		*Neisseria meningitidis*	4	2	2	5	4	4		3	2	4	4	3	4	4	2	2	2			2	3	2			4		2	2	4	3	3
84610	Mcbar-1351	Sweden		*Neisseria meningitidis*	4	2	2		4	4		3	2	4	4	3	4	4	2	2	2			12	3	2			4		2	2	4	3	3
645	204/92	Cuba	1992	*Neisseria meningitidis*	5	2	2	2	5	3		2	2	5	5	4	3	5	2	3	2				4	2			2		3	2	5	3	3
26036	70/92	Cuba	1992	*Neisseria meningitidis*	5	2	2	2	5	3		2	2	5	5	4	3	5	2	3	2				4	2			2		3	2	5	3	3
30311	NM8525	UK	2011	*Neisseria meningitidis*	4	2	2	6	3	4		3	2	7	5	6	4	5	4	4	3				5	2			4		2	2	4	3	3
31180	NM8525	UK	2011	*Neisseria meningitidis*	4	2	2	6	3	4		3	2	7	5	6	4	5	4	4	3				5	2			4		2	2	4	3	3
35273	10.1199.K	UK	2010	*Neisseria meningitidis*	4	2	2	20	3	4		3	2	7	5	6	4	5	4	4	3				5	2			4		2	2	4	3	3
35276	10.1516.S	UK	2010	*Neisseria meningitidis*	4	2	2	20	3	4		3	2	7	5	6	4	5	4	4	3				5	2			4		2	2	4	3	3
57442	24138	Ireland	2017	*Neisseria meningitidis*	4	2	2	20	3	4		3	2	29	5	6	4	41	4	4	3				5	2			4		2	2	4	3	3
3485	OX01061140	UK	2001	*Neisseria meningitidis*	2	2	2	10	3	4		3	2	4	5	9	3	11	2	5	4			2	6	2			4		2	3	4	3	3
10848	MK 89/96	Norway	1996	*Neisseria meningitidis*	2	2	2	10	3	4		3	2	4	5	9	3	14	2	5	4			2	6	2			4		2	3	4	3	3
10914	16044	Norway	1991	*Neisseria meningitidis*	2	2	2	10	3	4		3	2	4	5	9	3	14	2	5	4			2	6	2			4		2	3	4	3	3
28861	OX9930906	UK	1999	*Neisseria meningitidis*	2	2	2	10	3	4		3	2	4	5	9	3	14	2	5	4			2	6	2			4		2	3	4	3	3
28945	OX01061416	UK	2001	*Neisseria meningitidis*	2	2	2	10	3	4		3	2	4	5	9	3	11	2	5	4			2	6	2			4		2	3	4	3	3
56678	NLMB095	Canada	2012	*Neisseria meningitidis*	2	2	2	10	3	4		3	2	28	5	9	3	16	2	2	4			2	6	2					2	3	4	3	3
56722	NLMB019	Canada	2013	*Neisseria meningitidis*	2	2	2	10	3	4		3	2	28	5	33	3	16	2	2	4			2	6	2					2	3	4	3	3

id	isolate	country	year	species	NEIS2323 (virB1)	NEIS2324 (virB2)	NEIS2325 (virB3)	NEIS2326 (virB4)	NEIS2327 (virB5)	NEIS2328 (virB6)	NEIS2329 (virB7)	NEIS2330 (virB8)	NEIS2331 (virB9)	NEIS2332 (virB10)	NEIS2333 (virB11)	NEIS2334 (topA/topB)	NEIS2335 (T4CP/virBD4/traG)	NEIS2336	NEIS2337	NEIS2338 (relaxase/virBD2)	NEIS2339	NEIS2340 (fitB/stbB)	NEIS2341 (fitA/stbC)	NEIS2342	NEIS2343	NEIS2344	NEIS2345 (NgoΦ6 ORF10)	NEIS2347	NEIS2348	NEIS2349	NEIS2350 (trbM)	NEIS2351 (trbL)	NEIS2352 (traM)	NEIS2353	NEIS2354 (integrase)
20161	M10 240824	UK	2010	*Neisseria meningitidis*	6	3	2	11	3	6		6	4	8	6	10	10		5	6	5				7	3						2	6	3	6
21254	M11 241040	UK	2011	*Neisseria meningitidis*	6	3	2	15	3	6		6	4	8	6	10	10		5	6	5				7	3						2	6	3	6
28862	OX9930945	UK	1999	*Neisseria meningitidis*	6	3	2	11	3	6		6	4	8	6	10	10		5	6	5				7	3						2	6	3	6
34656	NM418	USA	2003	*Neisseria meningitidis*	6	3	2	11	3	6		6	4	8	6	10	10		5	6	5				7	3						2	6	3	6
34657	NM422	USA	2003	*Neisseria meningitidis*	6	3	2	11	3	6		6	4	8	6	10	10		5	6	5				7	3						2	6	3	6
35464	M13 240615	UK	2013	*Neisseria meningitidis*	6	3	2	15	3	6		6	4	8	6	10	10		5	6	5				7	3						2	6	3	6
35495	M13 240686	UK	2013	*Neisseria meningitidis*	6	3	2	11	3	6		6	4	8	6	10	10		5	6	5				7	3						2	6	3	6
39362	M15 240727	UK	2015	*Neisseria meningitidis*	6	3	2	11	3	6		6	4	8	6	10	10		5	6	5				7	3						2	6	3	6
39402	M15 240786	UK	2015	*Neisseria meningitidis*	6	3	2	23	3	6		6	4	8	6	10	10		5	6	5				7	3						2	6	3	6
42511	M16 240046	UK	2016	*Neisseria meningitidis*	6	3	2	11	3	6		6	4	8	6	10	10		5	6	5				7	3						2	6	3	6
20001	M10 240583	UK	2010	*Neisseria meningitidis*	6	3	2	11	3	6		6	4	9	6	11	10		5	6	5				7	3						2	6	3	6
20167	M11 240003	UK	2011	*Neisseria meningitidis*	6	3	2	11	3	6		6	4	8	6	14	10		5	6	5				7	3						2	6	3	6
21165	M11 240728	UK	2011	*Neisseria meningitidis*	6	3	2	11	3	6		6	4	8	6	14	10		5	6	5				7	3						2	6	3	6
26281	BB306	UK	2012	*Neisseria meningitidis*	6	3	2	11	3	6		6	4	8	6	16	13		5	6	5				7	3						2	6	3	6
26410	R306	UK	2011	*Neisseria meningitidis*	6	3	2	11	3	6		6	4	8	6	16	13		5	6	5				7	3						2	6	3	6
26416	T306	UK	2011	*Neisseria meningitidis*	6	3	2	11	3	6		6	4	8	6	16	13		5	6	5				7	3						2	6	3	6
26491	v306	UK	2012	*Neisseria meningitidis*	6	3	2	11	3	6		6	4	8	6	16	13		5	6	5				7	3						2	6	3	6
53331	M17 240170	UK	2017	*Neisseria meningitidis*	6	3	2	15	3	6		6	4	8	6	28	10		5	6	5				7	3						2	6	3	6
20013	M10 240604	UK	2010	*Neisseria meningitidis*	7	3	2	12	8	7		7	5	10	7	12	11	12	6	7	2				8	4						2	7	4	7
20453	M11 240434	UK	2011	*Neisseria meningitidis*	7	3	2	12	8	7		7	5	10	7	12	11	12	6	7	2				8	4						2	7	4	7
21098	M11 240451	UK	2011	*Neisseria meningitidis*	7	3	2	14	8	7		7	5	11	7	12	11	12	6	7	2				8	4						2	7	4	7
27978	M13 240166	UK	2013	*Neisseria meningitidis*	7	3	2	12	8	7		7	5	10	7	12	11	12	6	7	2				8	4						2	7	4	7
35297	11.1190.L	UK	2010	*Neisseria meningitidis*	7	3	2	12	8	7		7	5	10	7	12	11	12	6	7	2				8	4						2	7	4	7
35298	11.1286.D	UK	2011	*Neisseria meningitidis*	7	3	2	14	8	7		7	5	10	7	12	11	12	6	7	2				8	4						2	7	4	7
42594	M16 240162	UK	2016	*Neisseria meningitidis*	7	3	2	12	8	7		7	5	10	7	12	11	12	6	7	2				8	4						2	7	4	7
20139	M10 240790	UK	2010	*Neisseria meningitidis*	7	3	2	13	8	7		8	5	10	8	13	12	13	7	8	6				9	4						2	7	4	7
20218	M11 240059	UK	2011	*Neisseria meningitidis*	7	3	2	13	8	7		8	5	10	8	13	12	13	7	2	6				9	4						2	7	4	7
27516	21265	UK	2009	*Neisseria meningitidis*	7	3	2	13	8	7		8	5	10	8	13	12	13	7	2	6				9	4						2	7	4	7
39450	M07 240949	UK	2007	*Neisseria meningitidis*	7	3	2	13	8	7		8	5	10	8	13	12	13	7	2	6				9	4						2	7	4	7
53356	M17 240202	UK	2017	*Neisseria meningitidis*	7	3	2	13	8	7		8	5	10	8	13	21	13	7	2	6				9	4						2	7	4	7
57394	24058	Ireland	2017	*Neisseria meningitidis*	7	3	2		8	7		7	5	10	7	32		40	7		2				21	4						2		4	7
57510	24212	Ireland	2017	*Neisseria meningitidis*	7	3	2		8	7		7	5	10	7	32		40	7	7	2				21	4						2		4	7
27227	ERR049024	Unknown		*Neisseria gonorrhoeae*	8	4	5	16	6	9	2	10	7	30	10	17	6	15	10	10	13	3	2	5	11		9		7		7	6	2	2	8
31125	MU_NG1	Canada	1991	*Neisseria gonorrhoeae*	8	4	5	16	6	9	2	10	7	30	10	17	6	15	10	10	13	3	2	5	11		2		6		7	5	2	2	2
31562	WHO_F	Canada	1990	*Neisseria gonorrhoeae*	8	4	5	16	6	9	2	10	7	30	10	17	6	15	10	10	13	3	2	5	11		2		6		7	5	2	2	2
31970	02_230	Tanzania	1992	*Neisseria gonorrhoeae*	8	4	5	16	6	9	2	10	7	30	10	17	6	15	10	10	13	3	2	5	11		2		6		7	5	2	2	2
32028	02_232	Unknown		*Neisseria gonorrhoeae*	8	4	5	16	6	9	2	10	7	30	10	17	6	15	10	10	13	3	2	5	11		9		7		7	6	2	2	8
32049	DO7764	Unknown		*Neisseria gonorrhoeae*	8	4	5	16	6	9	2	10	7	30	10	17	9	19	10	13	13	3	2	5	11		2		7		7	7	2	2	8
32076	02_231	UK	2002	*Neisseria gonorrhoeae*	8	4	5	16	6	9	2	10	7	30	10	17	6	15	10	10	13	3	2	5	11		9		7		7	6	2	2	8
34770	ATL_2011_01_03	USA	2011	*Neisseria gonorrhoeae*	8	4	5	16	6	9	2	10	7	30	10	17	6	15	10	10	13	3	2	5	11		5		6		7	5	2	2	2
34790	MU_NG6	Australia	2001	*Neisseria gonorrhoeae*	8	4	5	16	6	9	2	10	7	30	10	17	6	15	10	10	13	3	2	5	11		2		6		7	5	2	2	2
37142	GCGS0353	USA	2000	*Neisseria gonorrhoeae*	8	4	5	16	6	9	2	10	7	30	10	17	6	15	10	10	13	3	2	5	11		9		6		7	5	2	2	2
37192	GCGS0454	USA	2001	*Neisseria gonorrhoeae*	8	4	5	16	6	9	2	10	7	30	10	17	6	15	10	10	13	3	2	5	11		9		6		7	5	2	2	2
37200	GCGS0479	USA	2012	*Neisseria gonorrhoeae*	8	4	5	16	6	9	2	10	7	30	10	17	6	15	10	10	13	3	2	5	11		5		6		7	5	2	2	2
37213	GCGS0257	USA	2000	*Neisseria gonorrhoeae*	8	4	5	16	6	9	2	10	7	30	10	17	6	15	10	10	13	3	2	5	11		9		6		7	5	2	2	2
45032	EXNG301 dup	Australia	2013	*Neisseria gonorrhoeae*	8	4	5	16	6	9	2	10	7	30	10	17	6	27	10	10	13	3	2	5	11		9				7	5	2	2	2
48789	SRR3360821	UK		*Neisseria gonorrhoeae*	8	4	5	16	6	9	2	10	7	30	10	17	6	15	10	10	13	3	2	5	11		2		6		7	5	2	2	2
48850	SRR3360882	UK		*Neisseria gonorrhoeae*	8	4	5	16	6	9	2	10	7	30	10	17	6	32	10	10	13	3	2	5	11		2		6		7	5	2	2	2
48858	SRR3360890	UK	2005	*Neisseria gonorrhoeae*	8	4	5	16	6	9	2	10	7	30	10	17	20	15	10	10	13	3	2	5	11		9		7		7	6	2	2	8
48897	SRR3360931	UK		*Neisseria gonorrhoeae*	8	4	5	16	6	9	2	10	7	30	10	17	6	15	10	10	13	3	2	5	11		2		6		7	5	2	2	2
32019	GC_12463	Unknown		*Neisseria gonorrhoeae*	8	4	5	16	6	9	2	10	7	30	10	20	6	15	10	10	13	3	2	5	11		5		6		7	5	2	2	2
48840	SRR3360872	UK	2004	*Neisseria gonorrhoeae*	8	4	5	16	6	9	2	10	7	30	10	20	6	15	10	10	13	3	2	5	11		5		6		7	5	2	2	2
48902	SRR3360936	UK	2004	*Neisseria gonorrhoeae*	8	4	5	16	6	9	2	10	7	30	10	20	6	15	10	10	13	3	2	5	11		5		6		7	5	2	2	2
48932	SRR3360967	UK	2004	*Neisseria gonorrhoeae*	8	4	5	16	6	9	2	10	7	30	10	20	6	15	10	10	13	3	2	5	11		5		6		7	5	2	2	2
48956	SRR3360991	UK	2004	*Neisseria gonorrhoeae*	8	4	5	16	6	9	2	10	7	30	10	20	6	15	10	10	13	3	2	5	11		5		6		7	5	2	2	2
8837	017-02	UK	1997	*Neisseria lactamica*	6	3	2	11	3	6		6	4	8	6	10	10		5	6	5	15			7	3						2	6	3	5

Table S1. Allele numbers corresponding to loci included in the PubMLST ‘VirB T4SS’ genomic island scheme. All isolates with ICE*riNK* variants present in sequencing data are listed. PubMLST assigns arbitrary allele numbers to each nucleotide variant of loci included in the scheme, and cells are coloured according to this allele number. *fitA*, *fitB*, and NgoΦ6 ORF10 of *N. gonorrhoeae* and *fitA* of *N. lactamica* are indicated by red boxes as these genes lie on chromosomal regions outside the ICE despite inclusion in this genomic island allele scheme.

Sequence (5'→3')	Dialect	*Neisseria gonorrhoeae* MS11	*Neisseria meningitidis* MC58	*Kingella negevensis* SW7208426	*Simonsiella muelleri* ATCC 29453	*Alysiella crassa* DSM 2578	*Neisseria gonorrhoeae* WHO F genomic island
ATGCCGTCTGAA	AT-DUS	1521	1477	4	2	4	3
AGGCCGTCTGAA	AG-DUS	190	214	1	3	0	0
AGGCAGCCTGAA	AG-kingDUS	14	19	1111	1940	3510	20
AGGCTGCCTGAA	AG-simDUS	15	16	906	1742	2450	11
AGGTCGTCTGAA	AG-mucDUS	75	88	0	8	0	0
AGGCTACCTGAA	AG-eikDUS	12	5	14	52	38	0
AAGCAGCCTGCA	AA-king3DUS	17	17	60	12	7	0
TGCCTGTCTGAA	TG-wadDUS	0	0	0	0	0	0
TTCAGGCAGCCTGAA	AG-king/sim dyad	7	7	796	1343	1887	11
	GC-content (%)	52.36	51.53	44.69	41.53	45.34	43.31

Table S2. Frequencies of exact DUS dialect sequence matches in various *Neisseriaceae* genomes and the WHO F 32 kb genomic islands. Cells are shaded in proportion to the relative abundance of each DUS dialect within a given genome. The DUS prevalence within the two identified WHO F genomic islands closely matches that of *K. negevensis*, *S. muelleri*, and *A. crassa*, indicating these species as possible donors of the island.

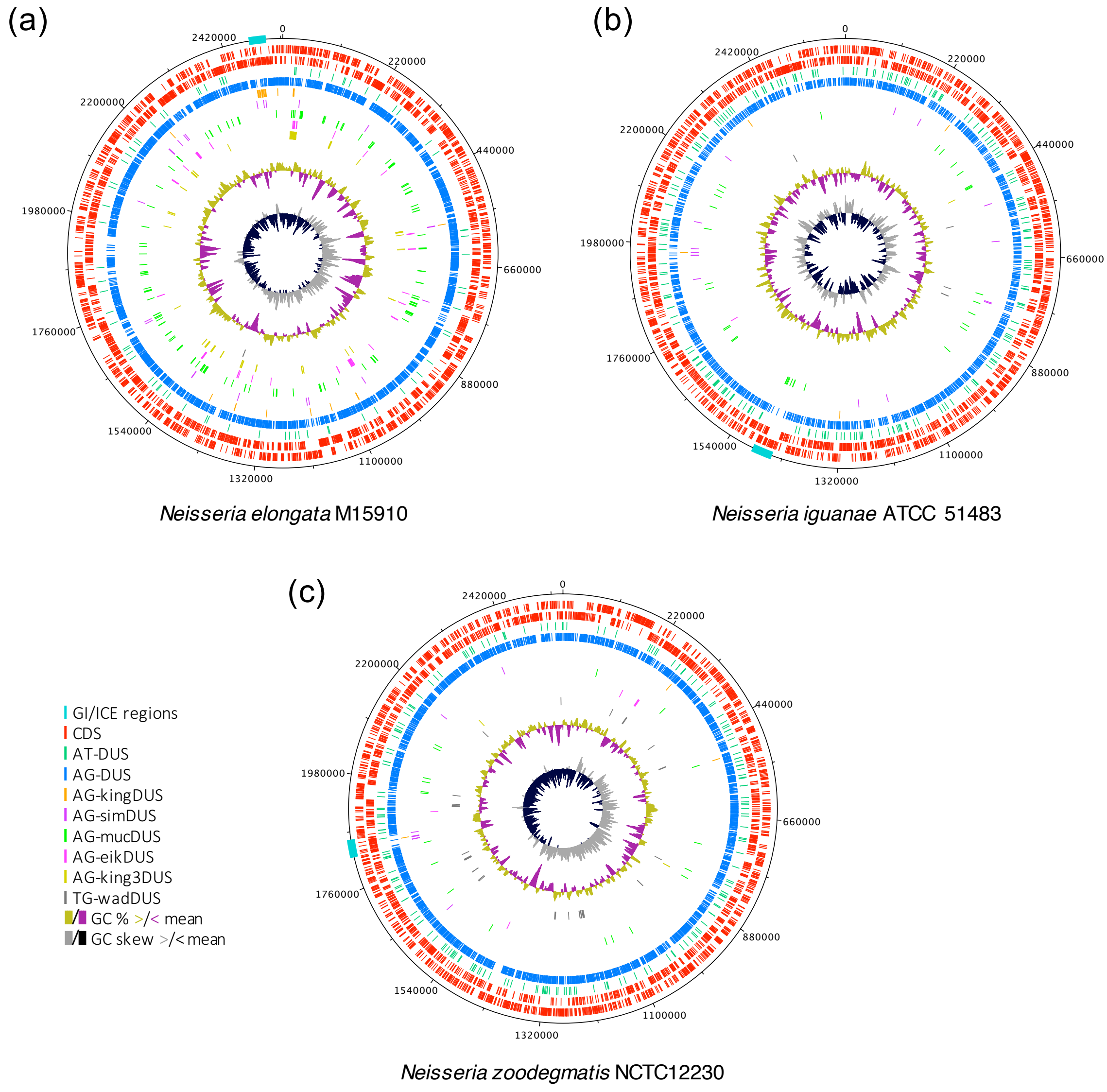

Figure S2. Further examples of ICE-like genomic islands in *Neisseria* genomes detected via their atypical DUS content. These GI regions (indicated by turquoise blocks in the outer ring) possess a T4SS operon, integrase, and accessory region in a similar arrangement to ICE*riNK* and ICE*Nmu*, and are located adjacent to a leucine (**a** & **b**) and asparagine (**c**) tRNA gene. Corresponding GenBank accession numbers are as follows: *N. elongata* M15910 – GCA_003351685.1, *N. iguanae* ATCC 51483 – GCA_003013245.1, *N. zoodegmatis.* NCTC12230 – GCA_900187305.1.
